# Associations between lifestyle factors, physiological conditions, and epigenetic age acceleration in an Asian population

**DOI:** 10.1007/s10522-025-10195-1

**Published:** 2025-02-05

**Authors:** Yu-Ru Wu, Wan-Yu Lin

**Affiliations:** 1 Institute of Epidemiology and Preventive Medicine, College of Public Health, National Taiwan University, Room 501, No. 17, Xu-Zhou Road, Taipei, 100 Taiwan; 2 Institute of Health Data Analytics and Statistics, College of Public Health, National Taiwan University, Taipei, Taiwan; 3 Master of Public Health Program, College of Public Health, National Taiwan University, Taipei, Taiwan

**Keywords:** Epigenetic clock, Aging, DNA methylation age, Oxidative stress, Biomarkers, Moking

## Abstract

Epigenetic clocks use DNA methylation (DNAm) levels to predict an individual’s biological age. However, relationships between lifestyle/biomarkers and epigenetic age acceleration (EAA) in Asian populations remain unknown. We here explored associations between lifestyle factors, physiological conditions, and epigenetic markers, including HannumEAA, IEAA, PhenoEAA, GrimEAA, DunedinPACE, DNAm-based smoking pack-years (DNAmPACKYRS), and DNAm plasminogen activator inhibitor 1 level (DNAmPAI1). A total of 2474 Taiwan Biobank (TWB) individuals aged between 30 and 70 provided physical health examinations, lifestyle questionnaire surveys, and blood and urine samples. Partial correlation analysis (while adjusting for chronological age, smoking, and drinking status) demonstrated that 29 factors were significantly correlated with at least one epigenetic marker (Pearson’s correlation coefficient |*r*|> 0.15). Subsequently, by exploring the model with the smallest Akaike information criterion (AIC), we identified the best model for each epigenetic marker. As a DNAm-based marker demonstrated to predict healthspan and lifespan with greater accuracy, GrimEAA was also found to be better explained by lifestyle factors and physiological conditions. Totally 15 factors explained 44.7% variability in GrimEAA, including sex, body mass index (BMI), waist-hip ratio (WHR), smoking, hemoglobin A1c (HbA1c), high-density lipoprotein cholesterol (HDL-C), creatinine, uric acid, gamma-glutamyl transferase (GGT), hemoglobin, and five cell-type proportions. In summary, smoking, elevated HbA1c, BMI, WHR, GGT, and uric acid were associated with more than one kind of EAA. At the same time, higher HDL-C and hemoglobin were related to epigenetic age deceleration (EAD). These findings offer valuable insights into biological aging.

**Supplementary Information:**

The online version contains supplementary material available at 10.1007/s10522-025-10195-1.

## Introduction

The global development of healthcare and medical technology has increased life expectancy. Many nations across the globe are witnessing a rise in the number and percentage of older people in their populations. By 2050, the 65-and-over population will increase to 38% in some countries (United Nations 2002). People with a slower aging rate will likely stay healthier in their declining years (McCrory et al. 2021). It was necessary to identify biomarkers to more accurately predict an individual’s aging rate (Baker and Sprott 1988). For over 30 years, scientists have explored biomarkers capable of predicting an individual’s biological age.

With the advancement of epigenetics in this decade, DNA methylation (DNAm) age has become a molecular measure of biological age (Horvath and Raj 2018). DNAm age is an aggregation of the DNAm levels at aging-related Cytosine-phosphate-Guanine (CpG) sites. For example, the first-generation epigenetic clocks referred to Hannum’s (Hannum et al. 2013) and Horvath’s clocks (Horvath 2013) based on 71 and 353 CpG sites, respectively. These 71 and 353 methylation sites were selected because of their superior predictive ability for chronological age. Moreover, incorporating DNAm levels from 513 and 1030 CpG sites, PhenoAge (Levine et al. 2018) and GrimAge (Lu et al. 2022, 2019) stood out as the second-generation DNAm-based aging clocks. They were developed to estimate the health span and lifespan.

GrimAge incorporated methylation-based smoking pack-years (DNAmPACKYRS) and seven plasma protein markers that were found to be linked to mortality and morbidity (Lu et al. 2022, 2019). GrimAge is a DNAm-based marker that has been demonstrated to predict healthspan and lifespan with greater accuracy (Lu et al. 2019). Among the eight GrimAge components (a DNAm-based estimator of smoking pack-years and seven DNAm surrogates), DNAm plasminogen activator inhibitor 1 (DNAmPAI1) outperforms GrimAge acceleration for several age-related traits. Being two components of GrimAge, DNAmPAI1 and DNAmPACKYRS warrant further investigation by predicting lifespan and time-to-coronary heart disease (Lu et al. 2019).

Recently, DunedinPACE (Dunedin Pace of Aging Calculated from the Epigenome) was developed and known as the third-generation epigenetic clock (Belsky et al. 2022). Belsky et al. (2022) analyzed the longitudinal data from ~ 1000 babies born during 1972–1973 in Dunedin, New Zealand, and they selected 173 CpG sites to predict individuals’ aging paces across ~ 20 years. DunedinPACE is an epigenetic marker aggregating the DNAm levels of these 173 CpG sites. It may predict a pace of aging beyond GrimAge (Belsky et al. 2020; 2022; Lin 2023).

In an era marked by a globally aging population, it is crucial to understand the intricate connections between lifestyle factors, biomarkers, and aging rate. Epigenetic age acceleration (EAA) is calculated as the residuals of regressing biological age on chronological age. Negative EAA implies biologically younger than people at the same chronological age. Conversely, positive EAA indicates biologically older than the counterparts. Multiple studies have highlighted that epigenetic aging is linked to physiological conditions. For example, EAA is related to cardiovascular health, fatty liver diseases, numerous cancer risks, cognitive function, respiratory organs, and kidney function (Fernanda et al. 2022; Lemke et al. 2022; Lu et al. 2019; Suarez et al. 2018; Wu et al. 2019; Yusipov et al. 2022).

Furthermore, much research has demonstrated a strong link between lifestyle-related factors and DNAm-based markers. Essential characteristics include dietary habits, alcohol consumption, educational attainment, physical activity, smoking, sleeping quality, body composition, and mental health status (Cardenas et al. 2022; Carroll et al. 2017; Carskadon et al. 2019; Crimmins et al. 2021; Han et al. 2019; Jansen et al. 2021; Kresovich et al. 2021; Quach et al. 2017; Sae-Lee et al. 2018; Wu et al. 2019; Xu et al. 2021; Zindler et al. 2022). These studies indicated that individuals may experience accelerated or decelerated epigenetic clocks due to these factors. For example, Kresovich et al. evaluated the associations of adiposity indicators and physical activity with four measures of EAA (HannumEAA, IEAA, PhenoEAA, and GrimEAA). Body mass index (BMI) and waist-hip ratio (WHR) were associated with most EAA measures, whereas physical activity was only associated with deceleration in GrimAge (p = 0.001) (Kresovich et al. 2021).

Several measures of EAA have been widely used to quantify biological aging. To investigate determinants that can influence the aging rate, we explore essential lifestyle factors and physiological conditions on seven DNAm-based markers in the Taiwanese population. Epigenetic age can predict health span and lifespan, which vary with ethnicity (Lu et al. 2019). For example, according to “Life Expectancy Estimates for 2022” reported by the U.S. Centers for Disease Control and Prevention (CDC), the average life expectancy for Asian Americans was 84.5 years, while it was 77.5 years for non-Hispanic whites (https://www.cdc.gov/nchs/data/vsrr/vsrr031.pdf). More studies on Asian populations will be necessary because most previous investigations have been based on individuals of European, African, or Hispanic ancestry (Belsky et al. 2022; Levine et al. 2018; Lu et al. 2022, 2019). In this work, we analyzed the DNAm data of 2474 Taiwan Biobank (TWB) individuals. A total of 81 factors were first screened through a partial correlation analysis. We then chose factors more correlated with any measure of EAA to perform a best-subset analysis. Through these procedures, we identified EAA-associated lifestyle factors and physiological conditions.

## Materials and methods

### The Taiwan biobank data

The TWB has collected health data from volunteers for investigation since 2012. This project recruited Taiwanese aged 30 to 70 without a cancer diagnosis history. After obtaining written informed consent, the TWB conducted comprehensive physical health examinations, blood and urine tests, and lifestyle questionnaire surveys for each individual. Trained and qualified health professionals were employed to assist participants in completing these items. Participants were required to provide 30 ml of venous blood, primarily for blood and genomic testing. Additionally, participants needed to provide a 15 ml urine sample for testing.

Height, weight, blood pressure levels, and lung function were all included in physical examinations. Standing height was gauged with a fixed stadiometer, and body weight was measured using an electronic load cell scale (Chen et al. 2024). TWB measured diastolic and systolic blood pressure (DBP, SBP) levels twice with a 5-min rest interval in a sitting position. Jamieson et al. suggested that two measurements of DBP (or SBP) should be taken, and the average of DBP (or SBP) should be recorded (Jamieson et al. 1990). Therefore, we averaged the two DBP (or SBP) measurements to analyze blood pressure more reliably. Details of lung function tests (spirometry) were described in a previous study investigating the TWB data (Chang et al. 2021).

The TWB questionnaire covered personal information, lifestyles, dietary habits, and environmental exposures. The questionnaire was designed by epidemiologists. Several workgroups evaluated the logical flows and clarity of the questions. Moreover, a pilot study was conducted to validate the questionnaire. The reliability of the questionnaire was measured by comparing the responses at baseline and follow-up visits (Feng et al. 2022). The blood and urine tests were carried out by laboratories certified with the ISO and College of American Pathologists (CAP) accreditation.

As of 2021, TWB randomly selected 2474 individuals for DNAm quantification. The 2474 subjects were randomly sampled from each county of Taiwan following the population sizes and male–female ratios. The DNAm levels were quantified through the Illumina Infinium MethylationEPIC BeadChip, encompassing approximately 860,000 CpG sites.

### Epigenetic age acceleration

We used the DNAm Age Calculator provided by Horvath’s laboratory, https://dnamage.genetics.ucla.edu/new, to calculate four measures of EAA and the two components of GrimEAA: HannumEAA (column “AgeAccelerationResidualHannum” from the DNAm Age Calculator output), IEAA (column “IEAA”), PhenoEAA (column “AgeAccelPheno”), GrimEAA (column “AgeAccelGrim2”, which was version 2 of GrimEAA (Lu et al. 2022)), DNAmPACKYRS (column “DNAmPACKYRS”), and DNAmPAI1 (column “DNAmPAI1”). We also used the R package “DunedinPACE” (https://github.com/danbelsky/DunedinPACE) to calculate DunedinPACE of each individual.

The quality control and normalization of the DNAm data were described in our previous work (Lin 2023). Specifically, to evaluate the quality of DNAm quantification for each sample, we calculated the average detection p-value across 27,526 CpGs used in the DNAm Age Calculator. Samples with more failed probes generally produce larger mean detection p-values. The average detection p-values of all 2474 samples were much smaller than the acceptable cutoff 0.01 (Maksimovic et al. 2016). Therefore, we regarded the quality of the TWB DNAm data as satisfactory.

After obtaining the seven epigenetic markers, we excluded extreme outliers from the subsequent analysis. Extreme outliers denoted values larger than ${Q}_{3}+3\times \left({Q}_{3}-{Q}_{1}\right)$ or smaller than ${Q}_{1}-3\times \left({Q}_{3}-{Q}_{1}\right)$, where ${Q}_{1}$ and ${Q}_{3}$ represented the first and third quartiles. Based on this criterion, we excluded 7, 1, 2, 5, 54, 0, and 1 extreme outliers for HannumEAA, IEAA, PhenoEAA, GrimEAA, DNAmPACKYRS, DNAmPAI1, and DunedinPACE, respectively. It is noteworthy that the former four EAA measures have been adjusted for chronological age (EAA is the residual of regressing the epigenetic age on chronological age), while the latter three markers have not.

Moreover, because the TWB collected the DNAm data from peripheral blood, an analysis while adjusting for cell-type composition is crucial. We used the Houseman deconvolution method (Houseman et al. 2012) to estimate five cell-type proportions, including B lymphocytes, natural killer cells, CD4^+^ T cells, CD8^+^ T cells, and monocytes.

### Lifestyle factors and physiological conditions

The 81 factors evaluated in our partial correlation analysis included four demographic variables, two obesity indicators (BMI and WHR), 15 lifestyle factors, 17 diet-related questions, 20 physiological conditions, 18 results for lung function tests, and the five cell-type proportions described in the previous section.

The four demographic variables included sex (male vs. female), chronological age (in years), educational attainment (an integer ranging from 1 to 7 representing different levels of education), and household composition (living alone vs. not living alone).

The 15 lifestyle factors included drinking (consuming more than 150 mL of alcoholic beverages per week, yes vs. no); active smoking (cigarette smoking for at least six months, yes vs. no); passive smoking (or secondhand smoking, yes vs. no); physical activities (exercising for at least 30 min thrice a week, yes vs. no); betel nut chewing (yes vs. no); regularly taking drugs such as cough syrup, sedatives, or pain relievers at least once a week (yes vs. no); regularly cooking meals by yourselves within 6 months before participating in the TWB (yes vs. no); being exposed to incense burning, mosquito coils, or fragrances for at least five minutes within the past year before joining the TWB (yes vs. no); consuming tea at least once daily (yes vs. no); coffee drinking thrice a week (yes vs. no); having a vegetarian diet for at least 6 months before joining the TWB (yes vs. no); the number of main meals per day (an integer ranging from 1 to 6); eating supper within an hour before bedtime (yes vs. no); eating out at least once in the past month when joining the TWB (yes vs. no); regularly taking vitamins, minerals, or supplements in the past month before joining the TWB (yes vs. no).

Furthermore, Supplementary Table S1 lists 17 diet-related questions in the TWB questionnaire. Each question described a dietary habit. For example, the first question was, “When you eat meat (such as pork, beef, mutton, chicken, duck, goose, etc.), do you eat it with fat, suet, or skin?” A participant was asked to choose one item from “Always,” “Most of the time,” “Half of the time,” “Seldom,” or “Never.” The five choices were scored as an integer ranging from 1 to 5.

A total of 20 physiological conditions contained SBP, DBP, heart rate, hemoglobin A1c (HbA1c), fasting glucose, total cholesterol, triglyceride, high-density lipoprotein cholesterol (HDL-C), low-density lipoprotein cholesterol (LDL-C), serum glutamic oxaloacetic transaminase, serum glutamic pyruvic transaminase, gamma-glutamyl transferase (GGT), total bilirubin, albumin, blood urea nitrogen (BUN), creatinine, uric acid, microalbumin, hemoglobin, and hematocrit.

Totally 18 results for lung function tests included vital capacity, vital capacity/height, forced vital capacity, forced expiratory volume in 1.0 s, (forced expiratory volume in 1.0 s/vital capacity)*100, forced expiratory volume in 1.0 s/forced vital capacity, forced expiratory volume in 1.0 s/predicted vital capacity, maximum mid-expiratory flow, peak expiratory flow, forced expiratory flow at 25%, forced expiratory flow at 50%, forced expiratory flow at 75%, forced expiratory flow at 75%/height, (Extrapolated Volume/forced vital capacity) *100, tidal volume, expiratory reserve volume, inspiratory reserve volume, and inspiratory capacity.

### Statistical analysis

We first conducted a partial correlation analysis to examine the correlation between lifestyles, physiological conditions, and epigenetic markers. Following Kawamura et al. (Kawamura et al. 2024), we adjusted the partial correlation analysis for chronological age, smoking, and drinking status. Because we plan to perform the best-subset analysis to select important factors from many explanatory variables, we hope to preserve more EAA-correlated factors that can be jointly considered in regression models. Therefore, we adopted a more liberal correlation cutoff ($\left|r\right|>$ 0.15) compared with Kawamura et al.’s criterion ($\left|r\right|>$ 0.20). Moreover, we calculated the 95% confidence interval (CI) for each partial correlation estimate (*r*). If $\left|r\right|>$ 0.15 and the 95% CI did not overlap with 0, we preserved the factor to the best-subset selection.

The R software (version 4.2.3) was used for our statistical analysis, and the R package **ppcor** (Kim 2015) was utilized for the partial correlation analysis. Subsequently, with the R package **bestglm** (https://cran.r-project.org/web/packages/bestglm/index.html), we performed the best-subset selection to investigate the optimal model for each epigenetic marker. We aimed to pinpoint a subset of explanatory variables (lifestyle factors and physiological conditions) that could best predict the outcome (epigenetic markers). We conducted an exhaustive search to achieve this goal while considering all possible combinations of the factors selected from the above partial correlation analysis. By exploring the model with the smallest Akaike information criterion (AIC), we identified the best model for each epigenetic marker.

The criterion to evaluate the best model for each epigenetic marker is the AIC, which is $-2\times \text{logLikelihood}+2p$ and *p* is the number of estimated parameters in the model (i.e., the number of predictors plus one intercept term). AIC strikes a balance between the ability of a model to describe the observed data and the number of parameters. A smaller AIC indicates that the model can depict the data well while following the principle of simplicity.

The false discovery rate (FDR) was computed according to the R built-in function p.adjust(p-vector, method = “BH”) (Benjamini and Hochberg 1995), where the p-vector contained p-values in the seven best models (each epigenetic marker had its own best model). We used the R package **car** (https://cran.r-project.org/web/packages/car/index.html) to compute the variance inflation factor (VIF). A VIF larger than 5 is an alarm of multicollinearity.

## Results

### Partial correlation analysis

The 2474 TWB individuals were randomly sampled from each county of Taiwan while considering the population sizes and male–female ratios. Over 99% of TWB subjects are Han Chinese, including Minnan Taiwanese, Hakka Taiwanese, and people of Chinese descent (Chen et al. 2016; Wei et al. 2021). To identify correlated factors with the seven DNAm-based markers, we conducted a partial correlation analysis for 81 factors, including blood biochemical measures, physical health examinations, and questionnaire surveys. Supplementary Figures S1-S10 revealed that each DNAm-based marker was correlated with several factors. With a partial correlation coefficient (*r*) cutoff of 0.15 (*r* > 0.15 or *r* < -0.15 regarded as correlated), the seven DNAm-based markers (HannumEAA, IEAA, PhenoEAA, GrimEAA, DNAmPACKYRS, DNAmPAI1, and DunedinPACE) were correlated with 4, 1, 4, 15, 15, 25, and 10 factors, respectively. GrimEAA (correlated with 15 factors) and its components, DNAmPAI1 (correlated with 25 factors) and DNAmPACKYRS (correlated with 15 factors), were related to more factors than other epigenetic clocks.

The liver function indicator (GGT) and the two kidney function measures (creatinine and uric acid) were “positively correlated” (*r* > 0 and the 95% CI did not overlap with 0) with four epigenetic markers (GrimEAA, DNAmPACKYRS, DNAmPAI1, and DunedinPACE), especially DNAmPAI1 (supplementary Figures S3-S4).

Most lifestyle-related factors were not correlated with any epigenetic markers (Figures S1 and S9), such as eating nuts (|*r*| $\le$ 0.03, Figure S1), playing sports (|*r*| $\le$ 0.07, Figure S1), taking drugs (|*r*| $\le$ 0.04, Figure S9), being exposed to incense (|*r*| $\le$ 0.05, Figure S9), drinking coffee (|*r*| $\le$ 0.08, Figure S9) or tea (|*r*| $\le$ 0.09, Figure S9), being a vegetarian (|*r*| $\le$ 0.04, Figure S9), eating supper (late-night snacks, |*r*| $\le$ 0.06, Figure S9), eating out at least once in the past month (|*r*| $\le$ 0.04, Figure S9), regularly taking supplements such as vitamins and minerals (|*r*| $\le$ 0.07, Figure S9), and the number of daily meals (|*r*| $\le$ 0.04, Figure S9).

If we treated $\left|r\right|>$ 0.15 and the 95% CI did not overlap with 0 as correlated, only two lifestyle factors were correlated with some epigenetic markers, including smoking (Figure S1) and cooking by yourselves (Figure S9). Smoking was positively correlated with four epigenetic markers, including DNAmPACKYRS (*r* = 0.61), GrimEAA (*r* = 0.38), DunedinPACE (*r* = 0.20), and DNAmPAI1 (*r* = 0.18). Cooking by yourselves within six months was negatively correlated with DNAmPAI1 (*r* = -0.22) and DNAmPACKYRS (*r* = -0.21, Figure S9). This result implies that smoking is an unhealthy habit. On the contrary, controlling things eaten into the body is beneficial by making food yourself.

Furthermore, the 17 items for diet preference were not correlated with any epigenetic marker (|*r*|< 0.15, Figure S8). Supplementary Table S1 lists the 17 diet-related questions in the TWB questionnaire. From diet question 1 (D1) to diet question 8 (D8), high points are healthier than low points according to common sense. For example, D1 asked, “When you eat meat (such as pork, beef, mutton, chicken, duck, goose, etc.), do you always (1 point)/never (5 points) eat it with fat, suet, or skin?” Conversely, low points are healthier than high points for D9-D17 (Supplementary Table S1). For example, D9 asked, “Do you always (1 point)/never (5 points) eat fruits or vegetables instead of high-fat snacks (such as chips, cakes, doughnuts, etc.) when enjoying snacks?” In line with our common sense, D1-D8 were generally negatively correlated with the seven epigenetic markers, while D9-D17 were roughly positively correlated with the epigenetic markers. This pattern can be observed in Figure S8.

In summary, given the partial correlation coefficient (*r*) cutoff of 0.15 (*r* > 0.15 or *r* < − 0.15 regarded as correlated), 29 out of the 81 factors were correlated with at least one epigenetic marker (listed in Table 1). The other 52 factors not correlated with any marker are listed in Supplementary Table S2. Among the 29 correlated factors, “cooking by yourselves” and six lung function measures (inspiratory capacity, inspiratory reserve volume, vital capacity, vital capacity/height, forced expiratory volume in 1.0 s, and forced vital capacity) were responded/measured in only ~ 60% of the 2,474 TWB individuals. If we put all 29 factors into a regression model, only the ~ 60% of individuals with complete data will be analyzed. Therefore, we considered the other 22 (= 29–7) characteristics as potential predictors. Besides, we did not perform multiple regression by modeling these 22 factors simultaneously to avoid multicollinearity. Instead, we put the 22 factors into the best-subset selection.

**Table 1:** Seven epigenetic markers and 29 EAA-correlated factors

	Males	Females	*P*-value ^a^
Total, n (%)	1243 (50.24)	1231 (49.76)	-
Chronological age (years), mean (s.d.)	50.24 (11.34)	49.25 (10.79)	0.025*
Seven epigenetic markers			
HannumEAA (years), mean (s.d.)	0.683 (3.57)	-0.733 (3.63)	< 0.001***
IEAA (years), mean (s.d.)	0.710 (3.78)	-0.735 (3.70)	< 0.001***
PhenoEAA (years), mean (s.d.)	0.069 (4.67)	-0.132 (5.00)	0.303
GrimEAA (years), mean (s.d.)	1.11 (4.00)	-1.19 (3.18)	< 0.001***
DNAmPACKYRS, mean (s.d.)	10.58 (8.94)	4.88 (5.07)	< 0.001***
DNAmPAI1 (pg/mL), mean (s.d.)	16,960 (2259.90)	14,655 (2207.51)	< 0.001***
DunedinPACE, mean (s.d.)	1.01 (0.11)	0.976 (0.10)	< 0.001***
Anthropometric indices			
Body mass index (kg/${m}^{2}$), mean (s.d.)	25.24 (3.43)	23.52 (3.72)	< 0.001***
Waist-hip ratio, mean (s.d.)	0.894 (0.06)	0.841 (0.07)	< 0.001***
Cardiovascular health metrics			
Systolic blood pressure (mmHg), mean (s.d.)	122.10 (16.35)	113.0 (17.13)	< 0.001***
Diastolic blood pressure (mmHg), mean (s.d.)	77.12 (10.62)	69.36 (10.44)	< 0.001***
Blood biochemical indicators			
Hemoglobin A1c (%), mean (s.d.)	5.77 (0.81)	5.66 (0.64)	< 0.001***
HDL-C (mg/dL), mean (s.d.)	48.51(11.58)	59.18 (13.61)	< 0.001***
Creatinine (mg/dL), mean (s.d.)	0.889 (0.18)	0.626 (0.40)	< 0.001***
Uric acid (mg/dL), mean (s.d.)	6.41 (1.33)	4.83 (1.08)	< 0.001***
GGT (U/L), mean (s.d.)	31.43 (45.35)	18.53 (15.60)	< 0.001***
Hematocrit (%), mean (s.d.)	45.95 (3.73)	40.79 (3.49)	< 0.001***
Hemoglobin (g/dL), mean (s.d.)	14.99 (1.19)	13.01 (1.19)	< 0.001***
Fasting glucose (mg/dL), mean (s.d.)	98.89 (22.07)	92.86 (17.10)	< 0.001***
Triglyceride (mg/dL), mean (s.d.)	134.32 (116.49)	101.73 (92.08)	< 0.001***
SGPT (U/L), mean (s.d.)	28.20 (22.25)	19.52 (12.51)	< 0.001***
Lung function measures			
Vital capacity (L), mean (s.d.)	3.76 (0.73)	2.63 (0.71)	< 0.001***
Vital capacity / Height (L/m), mean (s.d.)	2.20 (0.38)	1.66 (0.43)	< 0.001***
Forced vital capacity (L), mean (s.d.)	3.65 (0.72)	2.55 (0.75)	< 0.001***
Forced expiratory volume in 1.0 s (L), mean (s.d.)	2.66 (0.86)	1.85 (0.67)	< 0.001***
Inspiratory reserve volume (L), mean (s.d.)	1.58 (0.63)	0.986 (0.43)	< 0.001***
Inspiratory capacity (L), mean (s.d.)	2.58 (0.65)	1.74 (0.49)	< 0.001***
Lifestyle-related factors			
Smoking (yes vs. no) ^b^, n/a (%)	235/1243 (18.91%)	48/1231 (3.90%)	< 0.001***
Cooking by yourselves (yes vs. no) ^c^, n/a (%)	159/689 (23.08%)	549/752 (73.01%)	< 0.001***
Cell-type proportions			
B lymphocytes (%), mean (s.d.)	0.063 (0.03)	0.068 (0.03)	< 0.001***
Monocytes (%), mean (s.d.)	0.060 (0.02)	0.052 (0.02)	< 0.001***
Natural killer cells (%), mean (s.d.)	0.073 (0.05)	0.055 (0.05)	< 0.001***
CD4^+^ T cells (%), mean (s.d.)	0.128 (0.05)	0.144 (0.05)	< 0.001***
CD8^+^ T cells (%), mean (s.d.)	0.072 (0.04)	0.077 (0.04)	< 0.001***

Data are presented as mean (s.d.) or n/a (%) (n: the number of individuals belonging to this category; a: the total number of individuals responding to this question)

HDL-C, high-density lipoprotein cholesterol; GGT, gamma-glutamyl transferase; SGPT, serum glutamic pyruvic transaminase

*P*-value^a^: To test the difference between males and females, we used the two-sample t-test for continuous factors or the proportion test for dichotomous factors. If the difference is significant at p < 0.05, p < 0.01, or p < 0.001, we indicate it with *, **, or ***, respectively

Smoking^b^: individuals who had smoked cigarettes for at least six months when participating in the TWB

Cooking by yourselves^c^: individuals who cooked meals by themselves within six months when participating in the TWB

### Best-subset selection

While we aim to explore the association between lifestyle factors, physiological conditions, and EAA, we cannot put all 22 factors into the prediction model due to multicollinearity (i.e., VIF > 5). Therefore, we explored the best model with the smallest AIC for each epigenetic marker, indicating that the model had the best performance while accounting for the model complexity. However, $\text{loglikelihood}$ changes with the response variable (i.e., epigenetic marker), $\text{AIC}=-2\times \text{logLikelihood}+2p$ (where *p* is the number of estimated parameters) cannot be compared across different epigenetic markers. Hence, we used the adjusted R-square to evaluate the explanatory ability of the seven best models.

Unlike the conventional R-square, the adjusted R-square can compare models with different numbers of predictors. Through the best-subset analysis, the adjusted R-squares for the seven DNAm markers were ranked as (supplementary Table S3): DNAmPACKYRS (53.5%) > GrimEAA (44.7%) > DNAmPAI1 (42.2%) > DunedinPACE (29.1%) > PhenoEAA (19.9%) > HannumEAA (19.3%) > IEAA (5.6%). GrimEAA and its two components (DNAmPACKYRS and DNAmPAI1) can be best explained by the predictors, followed by DunedinPACE.

Although DNAmPACKYRS had the highest adjusted R-square (53.5%) among all DNAm-based markers, it is one of the eight components belonging to GrimAge. Because our primary purpose was to investigate factors associated with the aging rate, we chose GrimEAA’s model for further interpretation. The best model for GrimEAA includes 15 explanatory variables: sex, BMI, WHR, smoking, hemoglobin A1c (HbA1c), HDL-C, creatinine, uric acid, GGT, hemoglobin, B lymphocytes, natural killer cells, CD4^+^ T cells, CD8^+^ T cells, and monocytes. The three indicators of kidney (creatinine, uric acid) and liver (GGT) function were all selected as predictors for GrimEAA.

Following the partial correlation analysis, GrimEAA and DNAmPAI1 were correlated with more factors (15 and 25, respectively). Therefore, we put the best model for GrimEAA and DNAmPAI1 in Table 2, and those for the remaining five DNAm-based markers are presented in Supplementary Table S4. All VIF values were under 5 when considering the factors in the best model as the explanatory variables. If we bypass the best-subset analysis and directly put all 22 factors into a regression model, some VIF values will be larger than 5. Therefore, refining models through the best-subset selection is justifiable.

**Table 2:** The best model for GrimEAA and DNAmPAI1

	$\beta$	Standard error	95% Confidence interval		VIF	FDR ^a^
GrimEAA (in years)						
Sex (female vs. male)	− 0.7601	0.1761	[− 1.1055,	− 0.4148]	2.4042	4.0E-05***
BMI (kg/${m}^{2}$)	0.0658	0.0198	[0.0269,	0.1047]	1.6489	1.8E-03**
WHR	3.8404	1.1057	[1.6722,	6.0087]	1.7348	1.0E-03**
CD8^+^ T cells (%)	− 12.8228	1.3528	[− 15.4755,	− 10.1701]	1.0584	4.3E-20***
CD4^+^ T cells (%)	− 10.9774	1.2197	[− 13.3692,	− 8.5857]	1.3668	3.0E-18***
Natural killer cells (%)	− 9.9934	1.2094	[− 12.3650,	− 7.6218]	1.1125	1.3E-15***
B lymphocytes (%)	− 21.1413	2.4250	[− 25.8966,	− 16.3860]	1.2925	3.3E-17***
Monocytes (%)	14.7432	2.8452	[9.1638,	20.3225]	1.1923	7.1E-07***
HbA1c (%)	0.5608	0.0826	[0.3988,	0.7227]	1.1333	6.4E-11***
HDL-C (mg/dL)	− 0.0149	0.0050	[− 0.0246,	− 0.0051]	1.4388	4.6E-03**
Hemoglobin (g/dL)	− 0.2383	0.0492	[− 0.3348,	− 0.1417]	1.7987	3.7E-06***
GGT (U/L)	0.0078	0.0017	[0.0044,	0.0112]	1.1190	1.7E-05***
Creatinine (mg/dL)	0.4178	0.1873	[0.0504,	0.7851]	1.2428	3.2E-02*
Uric acid (mg/dL)	0.1247	0.0524	[0.0219,	0.2275]	1.7801	2.3E-02*
Smoking (yes vs. no)	5.1756	0.1923	[4.7985,	5.5527]	1.1500	2.6E-138***
**DNAmPAI1 (in pg/mL)**						
Chronological age (years)	36.4324	4.3211	[27.9590,	44.9057]	1.5518	3.4E-16***
Sex (female vs. male)	− 1227.8125	103.1327	[− 1430.0486,	− 1025.5764]	1.8009	9.8E-31***
BMI (kg/${m}^{2}$)	76.2614	13.8109	[49.1793,	103.3436]	1.7454	1.4E-07***
WHR	1969.7495	783.5495	[433.2635,	3506.2356]	1.9005	1.6E-02*
CD8^+^ T cells (%)	− 7210.6949	946.2652	[− 9066.2550,	− 5355.1348]	1.1329	1.9E-13***
Natural killer cells (%)	− 3156.9640	861.0496	[− 4845.4223,	− 1468.5056]	1.2326	5.1E-04***
B lymphocytes (%)	− 7049.4816	1533.4944	[− 10056.5573,	− 4042.4059]	1.1341	1.1E-05***
Monocytes (%)	4858.5363	1848.9684	[1232.8381,	8484.2345]	1.0991	1.2E-02*
SBP (mmHg)	3.9715	2.6354	[− 1.1965,	9.1394]	1.4139	1.3E-01
HbA1c (%)	293.6134	94.1388	[109.0137,	478.2130]	3.2110	3.3E-03**
Fasting glucose (mg/dL)	8.7013	3.4118	[2.0109,	15.3916]	3.1472	1.5E-02*
Triglyceride (mg/dL)	1.9987	0.4234	[1.1685,	2.8290]	1.3717	6.4E-06***
HDL-C (mg/dL)	− 9.4294	3.5275	[− 16.3465,	− 2.5122]	1.5842	1.1E-02*
GGT (U/L)	4.4551	1.1889	[2.1238,	6.7864]	1.1445	3.8E-04***
Uric acid (mg/dL)	189.0567	34.8675	[120.6840,	257.4294]	1.7299	2.1E-07***
Smoking (yes vs. no)	701.5314	128.5739	[449.4070,	953.6558]	1.1346	1.8E-07***

*VIF* variance inflation factor, *FDR* false discovery rate, *BMI* body mass index, *WHR* waist-hip ratio, *HbA1c* hemoglobin A1c, *HDL-C* high-density lipoprotein cholesterol, *GGT* gamma-glutamyl transferase

FDR^a^: Statistical significance is marked with *, **, and ***, representing an FDR less than 0.05, 0.01, and 0.001, respectively

**Fig. 1:**
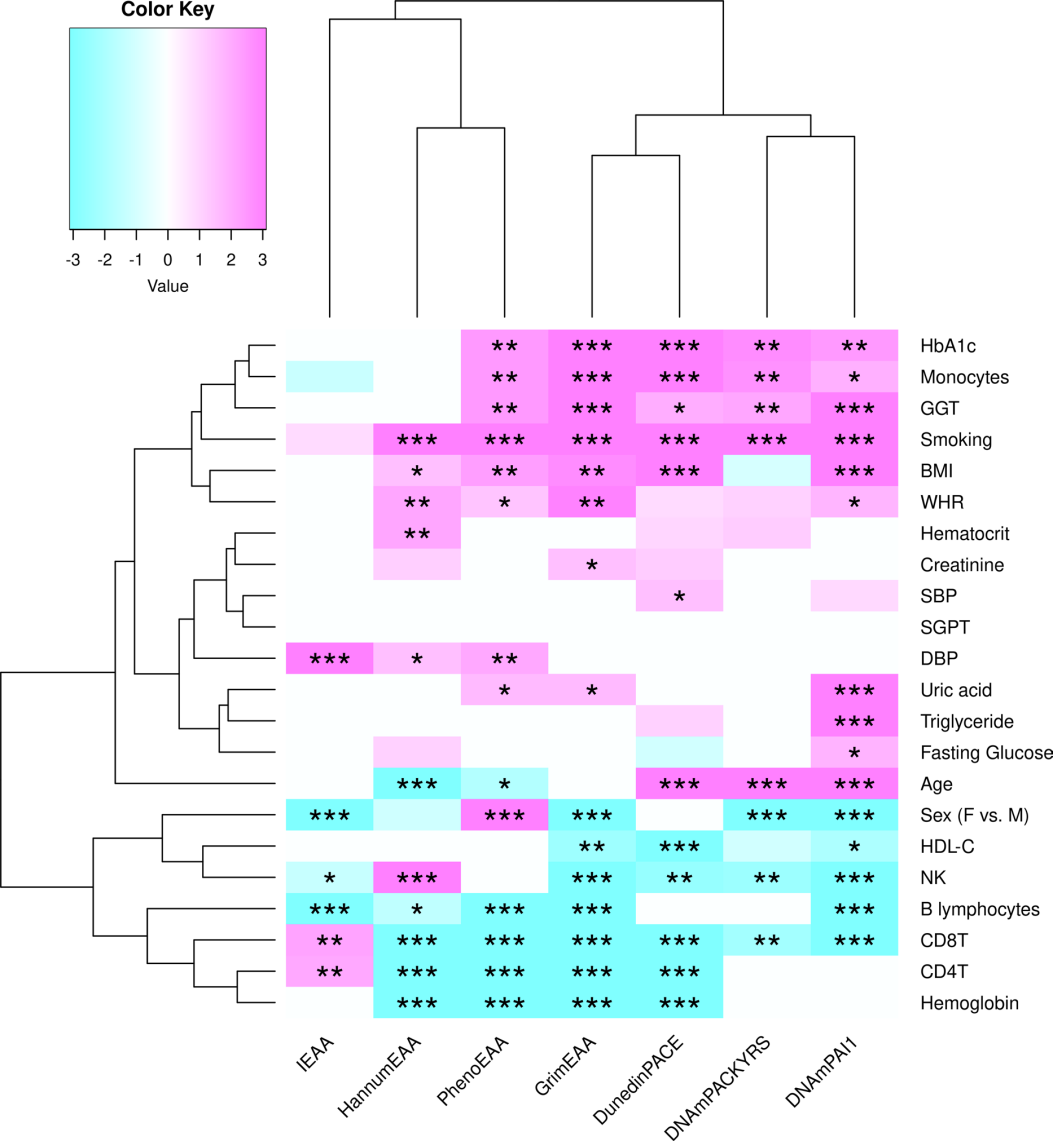
The phylogenetic heat map of the factor-EAA associations. This figure summarizes the results from Table 2 and Supplementary Table S4. The magnitude of the value represents –log_10_(FDR), which is always positive. Moreover, we deliberately added a positive/negative sign before the magnitude. A positive/negative sign indicates that the regression coefficient ($\beta$) is positive/negative. Statistical significance is marked with *, **, and ***, representing an FDR less than 0.05, 0.01, and 0.001, respectively.

We summarized the factor-EAA associations in a phylogenetic heat map (Fig. 1). The magnitude of the value represents –log_10_(FDR), which is always positive. Moreover, we deliberately added a positive/negative sign before the magnitude. A positive/negative sign indicates that the regression coefficient (β) is positive/negative. As shown in Fig. 1, the 22 factors can be roughly categorized as the top (red) and bottom (blue) parts. A total of 96 p-values were put into the R built-in function p.adjust(p-vector, method = “BH”), where “BH” indicated the Benjamini–Hochberg method (Benjamini and Hochberg 1995), where 96 was the total number of factors in the seven best models (96 checks in Table S3).

Being a female, having higher HDL-C and hemoglobin levels was associated with epigenetic age deceleration (EAD), shown as blue cells in Fig. 1. On the contrary, smoking, higher BMI, WHR, HbA1c, GGT, uric acid, creatinine, and triglyceride were all positively associated with EAA, demonstrated as red cells in Fig. 1. The details of Fig. 1 can be found in Table 2 and Supplementary Table S4.

## Discussion

Two systematic reviews, including 156 and 299 publications, respectively, have revealed that EAA is associated with smoking, a larger BMI, male sex, and diabetes (Chervova et al. 2024; Oblak et al. 2021). These conclusions align with our work (Fig. 1). By investigating DNAm samples from 144 Japanese men aged 65–72 years, Kawamura et al. found that the contribution (adjusted R-square) of 16 factors was 5.6% for PhenoEAA and 32.9% for GrimEAA (Kawamura et al. 2024). Their 16 factors included oxygen uptake at the ventilatory threshold (physical fitness), peak oxygen uptake (physical fitness), grip strength (physical fitness), fat-free mass (anthropometric factor), visceral fat area (anthropometric factor), calf circumference (anthropometric factor), head fat percentage, triglyceride, HDL-C, carbohydrate intake (nutrient), copper intake (nutrient), vitamin C intake (nutrient), beta-carotene intake (nutrient), smoking, drinking, and chronotype.

In this work, we used 14 and 15 factors to construct the PhenoEAA and GrimEAA models, respectively contributing 19.9% and 44.7% of the adjusted R-square (Table S3). Although the sample size (2474 vs. 144), study population (Taiwanese vs. Japanese), and age range (30–70 vs. 65–72) are different, this comparison may imply that our factors are more relevant to PhenoEAA and GrimEAA than those investigated in Kawamura et al.’s research (Kawamura et al. 2024).

The TWB data showed the relationship between epigenetic aging, lifestyle factors, and physiological conditions. This work revealed some novel findings. **Firstly**, the EAA that can be mostly explained by lifestyle, kidney or liver function, and metabolic factors is GrimEAA (Adjusted R-square = 44.7%, Table S3). By contrast, IEAA is the measure least explained by the factors in this study (Adjusted R-square = 5.6%, Table S3). **Secondly**, elevated GGT, creatinine, and uric acid are associated with an increased risk of liver (GGT) and kidney (creatinine and uric acid) impairment (Cho et al. 2023; Joo et al. 2020). Our result shows that GrimEAA can better reflect these biomarkers (Fig. 1). **Thirdly**, smoking was identified as the primary lifestyle factor significantly contributing to biological aging, as evidenced by its strong associations with all epigenetic markers except for IEAA (Fig. 1). On the other hand, secondhand smoking (or passive smoking) was not related to any EAA (Figure S1).

**Obesity** traits such as BMI and WHR were associated with various measures of EAA, except for IEAA and DNAmPACKYRS (Fig. 1). This result indicates that general (represented by BMI) and abdominal obesity (measured by WHR) are independent risk factors of aging acceleration. We could incorporate both indices into a regression model without the multicollinearity problem (VIF < 5, Tables 2 and S4).

Four **lipid traits** were investigated in this work, including HDL-C, LDL-C, triglyceride, and total cholesterol. Only HDL-C and triglyceride were correlated (Figure S2, $\left|r\right|>$ 0.15) and associated (Fig. 1) with epigenetic markers. As reported by other studies, these two lipid traits are more critical to coronary heart disease, and the triglyceride/HDL-C ratio is a useful indicator for detecting metabolic syndrome (Borrayo et al. 2018). DNAmPAI1 is the only epigenetic marker associated with triglyceride (FDR < 0.001, Fig. 1). This result aligns with European ancestry data—DNAmPAI1 stands out regarding the association with triglyceride levels (Lu et al. 2019).

Except for smoking, most lifestyle factors were not related to EAA ($\left|r\right|\le$ 0.15), such as education, living alone, performing physical exercise (Figure S1), drinking tea or coffee, vegetarian eating style, regularly taking vitamins (Figure S9), etc. Although dietary habits were not prominently correlated with any epigenetic marker (D1-D17, $\left|r\right|\le$ 0.15, Figure S8), the pattern in Figure S8 suggested that a healthier dietary attitude was slightly related to EAD. Examples included “Never eat fat, suet, or skin when eating meat (such as pork, beef, mutton, chicken, duck, goose, etc.)” and “Always eat fruits or vegetables instead of high-fat snacks (such as chips, cakes, doughnuts, etc.) when enjoying snacks” (D1-D17 dietary habits can be found in Supplementary Table S1).

Typically, **hemoglobin** levels decline with aging. Studies have shown an increasing prevalence of anemia in older people (Le 2016). Our results show that a low hemoglobin level is associated with all measures of EAA (FDR < 0.001, the bottom row of Fig. 1), except for IEAA.

This study included three **kidney** function indicators: creatinine, uric acid, and BUN. BUN was not correlated with any epigenetic marker ($\left|r\right|\le$ 0.15, Figure S4). Creatinine and uric acid were correlated with GrimEAA and its components (DNAmPAI1 and DNAmPACKYRS) ($\left|r\right|>$ 0.15, Figure S4). When considering the 15 factors selected by GrimEAA’s best model, creatinine and uric acid were still associated with GrimEAA (FDR < 0.05, Fig. 1). Uric acid was even more associated with GrimEAA’s component—DNAmPAI1 (FDR < 0.001, Fig. 1). PAI1 and uric acid are both critical factors in blood clotting and metabolic abnormalities (Jin et al. 2012; Zhang et al. 2020). As the epigenetic surrogate marker of PAI1, DNAmPAI1 is inherently the DNAm marker most related to uric acid.

Three measures of **liver** function were considered in this work: total bilirubin, albumin, and GGT. Total bilirubin and albumin were not correlated with any epigenetic marker ($\left|r\right|\le$ 0.15, Figure S3). GGT was correlated with DNAmPAI1 ($\left|r\right|>$ 0.15, Figure S3). A Korean nationwide database including 9.6 million individuals showed that higher GGT levels were associated with increased mortality in cardiovascular disease, cancer, respiratory disease, and liver disease (Cho et al. 2023). This study implies that GGT is critical to human health and not only restricted to the liver. As shown by Fig. 1, GGT was associated with all epigenetic markers except for the first-generation clocks (HannumEAA and IEAA). This evidence confirms that a worse liver function is related to acceleration in epigenetic age assessed by the second or third-generation clocks.

Two **diabetes** traits, fasting glucose and HbA1c, were investigated here. Like GGT, HbA1c was associated with all epigenetic markers except for the first-generation clocks (HannumEAA and IEAA, Fig. 1). Fasting glucose was not as relevant to EAA as HbA1c (Fig. 1). A possible explanation for this finding is that HbA1c measures blood sugar for the recent 2 ~ 3 months; consequently, it is less sensitive to acute hyperglycemic changes than fasting glucose. HbA1c is regarded as a more reliable measure of long-term glycemic levels than fasting glucose (Sherwani et al. 2016).

The main conclusions have been summarized in Fig. 1. From the findings of this work, people may slow their aging rate by controlling the HbA1c, BMI, WHR, GGT, uric acid, creatinine, triglyceride, avoiding smoking, and preventing a low HDL-C or a low hemoglobin level.

The biological aging rate is associated with lifestyle factors and physiological conditions. With a sizable sample size (2474), we built the best model for each of the seven commonly discussed epigenetic markers. The main limitation of this study is the incomplete survey of some factors. To save time, ~ 40% of the participants selected simplified questionnaires and examinations. Although 29 factors passed the partial correlation filter, “cooking by yourselves” and six lung function measures were responded/measured in only ~ 60% of the 2474 TWB individuals. Therefore, we had to build the best model with the remaining 22 factors.

## Conclusion

The adjusted R-squares for the five measures of EAA were ranked as (supplementary Table S3): GrimEAA (44.7%) > DunedinPACE (29.1%) > PhenoEAA (19.9%) > HannumEAA (19.3%) > IEAA (5.6%). GrimEAA can be best explained by physiological conditions and lifestyle factors, while IEAA is difficult to explain. The result revealed that three liver or kidney function indicators (GGT, creatinine, and uric acid) were positively associated with GrimEAA (Fig. 1). Our research explored the connections between DNAm-based markers, liver or kidney functions, and lifestyle-related factors, particularly for the Asian population.

## Supplementary Information

Below is the link to the electronic supplementary material.

Supplementary file1 (DOCX 1458 KB)

## Data Availability

The participant-relevant data supporting the findings of this study is made available by the Taiwan Biobank. To access this data, applicants should submit a request through the official website: https://www.twbiobank.org.tw/.
