# Supplementary material for: Associations between lifestyle factors, physiological conditions, and epigenetic age acceleration in an Asian population
Source: Biogerontology. 2025 Feb 5;26(2):51. doi: 10.1007/s10522-025-10195-1 (PMC11799100; doi:10.1007/s10522-025-10195-1)
Supplement: Supplementary file 1 — Supplementary file1 (DOCX 1458 KB) [file 10522_2025_10195_MOESM1_ESM.docx]

Supplementary Materials

# Associations between Lifestyle Factors, Physiological Conditions, and Epigenetic Age Acceleration in an Asian Population

Yu-Ru Wu ^1^, Wan-Yu Lin ^1,2,3^*

^1^Institute of Epidemiology and Preventive Medicine, College of Public Health, National Taiwan University, Taipei, Taiwan

^2^Institute of Health Data Analytics and Statistics, College of Public Health, National Taiwan University, Taipei, Taiwan

^3^Master of Public Health Program, College of Public Health, National Taiwan University, Taipei, Taiwan

Running title: Lifestyle factors, physiological conditions, and epigenetic age acceleration

Yu-Ru Wu (<https://orcid.org/0009-0007-4396-0844>)

Wan-Yu Lin, Ph.D.(<http://orcid.org/0000-0002-3385-4702>)

* Corresponding author: Wan-Yu Lin, Ph.D.

Room 501, No. 17, Xu-Zhou Road, Taipei 100, Taiwan

Phone/Fax: +886-2-33668106

E-mail: linwy@ntu.edu.tw

Contents

[Associations between Lifestyle Factors, Physiological Conditions, and Epigenetic Age Acceleration in an Asian Population 1](#_Toc188456726)

[Tables 3](#_Toc188456727)

[Table S1 The 17 diet-related questions in the TWB questionnaire. 3](#_Toc188456728)

[Table S2 Fifty-two factors not correlated with any epigenetic marker 5](#_Toc188456729)

[Table S3 Best-subset selection for 7 DNA methylation-based markers (22 factors passing the partial correlation filtering serve as candidate predictors) 8](#_Toc188456730)

[Table S4 The best model for HannumEAA, IEAA, PhenoEAA, DNAmPACKYRS, and DunedinPACE 9](#_Toc188456731)

[Figures 12](#_Toc188456732)

[Figure S1 Partial correlation analysis between DNAm-based markers and lifestyle factors. 12](#_Toc188456733)

[Figure S2 Partial correlation analysis between DNAm-based markers, cardiovascular health metrics, and blood biochemical indicators 13](#_Toc188456734)

[Figure S3 Partial correlation analysis between DNAm-based markers and blood biochemical indicators. 14](#_Toc188456735)

[Figure S4 Partial correlation analysis between DNAm-based markers and blood biochemical indicators 15](#_Toc188456736)

[Figure S5 Partial correlation analysis between DNAm-based markers, lung function measures, and blood biochemical indicators 16](#_Toc188456737)

[Figure S6 Partial correlation analysis between DNAm-based markers and lung function measures 17](#_Toc188456738)

[Figure S7 Partial correlation analysis between DNAm-based markers and lung function measures. 17](#_Toc188456739)

[Figure S8 Partial correlation analysis between DNAm-based markers and diet preference 18](#_Toc188456740)

[Figure S9 Partial correlation analysis between DNAm-based markers and lifestyle-related factors 18](#_Toc188456741)

[Figure S10 Partial correlation analysis between DNAm-based markers and cell-type proportions 19](#_Toc188456742)

[References: 19](#_Toc188456743)

# Tables

# Table S1 The 17 diet-related questions in the TWB questionnaire.

| Dietary Habits/ Food Category | Always | Most of the time | Half of the time | Seldom | Never |
| --- | --- | --- | --- | --- | --- |
| 1. When you eat meat (such as pork, beef, mutton, chicken, duck, goose, etc.), do you eat it with fat, suet, or skin? | 1 | 2 | 3 | 4 | 5 |
| 1. When you eat fish or meat, do you prefer cooking it with oil (such as frying, deep-frying, frying, braising, steamed fish topped with oil, etc.)? | 1 | 2 | 3 | 4 | 5 |
| 1. When you eat vegetables, do you prefer cooking them in a stir-fry way? | 1 | 2 | 3 | 4 | 5 |
| 1. When you eat rice or noodles (staple food), do you eat them with marinade, gravy, or lard? | 1 | 2 | 3 | 4 | 5 |
| 1. When you eat soy foods, do you prefer cooking them in a deep-fry way (such as fried tofu, stinky tofu, fried tofu skin, etc.)? | 1 | 2 | 3 | 4 | 5 |
| 1. When you eat bread, do you spread butter, plant-based butter (margarine), or mayonnaise? | 1 | 2 | 3 | 4 | 5 |
| 1. When you have a meal, do you add additional salt, soy sauce, chili sauce, or any other seasoning? | 1 | 2 | 3 | 4 | 5 |
| 1. Are you used to having pickles, fermented tofu, and fermented soybeans as side dishes in a meal? | 1 | 2 | 3 | 4 | 5 |
| 1. Do you eat fruits or vegetables instead of high-fat snacks (such as chips, cakes, doughnuts, etc.) when you have snacks? | 1 | 2 | 3 | 4 | 5 |
| 1. When you prepare meat (such as pork, beef, mutton, chicken, duck, goose, etc.) for a meal, do you cook it roasted or braised instead of deep-frying? | 1 | 2 | 3 | 4 | 5 |
| 1. If a food product has a low-fat option (such as low-fat ice cream, low-fat milk, skim milk, low-fat salad sauce, etc.), would you choose it instead of a regular product? | 1 | 2 | 3 | 4 | 5 |
| 1. Do you eat food with low-sodium ingredients (such as low-sodium salt, lower-sodium soy sauce, etc.)? | 1 | 2 | 3 | 4 | 5 |
| 1. Would you like to eat lower-fat meat (such as fish or chicken) instead of higher-fat meat (such as beef or pork)? | 1 | 2 | 3 | 4 | 5 |
| 1. Would you choose to eat lean meat instead of fatty meat? | 1 | 2 | 3 | 4 | 5 |
| 1. Would you choose to perform a vegetarian and light diet in certain meals to reduce the intake of higher-fat food such as meat or fat? | 1 | 2 | 3 | 4 | 5 |
| 1. Do you eat at least two kinds of vegetables a day? | 1 | 2 | 3 | 4 | 5 |
| 1. When you have meat, do you intentionally eat less? | 1 | 2 | 3 | 4 | 5 |

This table was Supplementary Table S4 in Lo and Lin (Lo & Lin, 2022).

#

# Table S2 Fifty-two factors not correlated with any epigenetic marker

|  | **Males** | **Females** |
| --- | --- | --- |
| Total, n | 1243 | 1231 |
| Educational attainment ^a^ (1-7 degree level) | 5.75 (0.86) | 5.40 (0.95) |
| Living alone (yes vs. no), n/a (%) | 90/1243 (7.24%) | 140/1231 (11.37%) |
| **Heart-related factors** |  |  |
| Heart rate (beats per 30 seconds) | 34.76 (4.83) | 35.07 (4.15) |
| **Blood biochemical indicators** |  |  |
| LDL-C (low-density lipoprotein cholesterol) (mg/dL) | 122.42 (31.49) | 120.42 (33.36) |
| Total cholesterol (mg/dL) | 192.20 (34.17) | 197.30 (35.94) |
| SGOT (serum glutamic oxaloacetic transaminase) (U/L) | 26.03 (11.02) | 22.67 (7.64) |
| Total bilirubin (mg/dL) | 0.750 (0.31) | 0.619 (0.24) |
| Albumin (g/dL) | 4.61 (0.25) | 4.52 (0.25) |
| Blood urea nitrogen (mg/dL) | 14.01 (3.68) | 12.59 (4.29) |
| Microalbumin (mg/L) | 32.25 (141.45) | 22.70 (89.40) |
| **Lung function measures** (FEV1: forced expiratory volume in 1.0 second) | | |
| FEV1 / Forced vital capacity (%) | 72.83 (18.12) | 72.25 (18.07) |
| FEV1 / Vital capacity (%) | 70.75 (18.53) | 70.99 (20.26) |
| FEV1 / predicted vital capacity (%) | 70.92 (21.21) | 69.29 (23.39) |
| Maximum mid-expiratory flow (L/sec) | 2.78 (1.34) | 1.92 (0.97) |
| Peak expiratory flow (L/sec) | 4.75 (2.59) | 3.09 (1.71) |
| Forced expiratory flow at 25% (L/sec) | 4.41 (2.45) | 2.92 (1.67) |
| Forced expiratory flow at 50% (L/sec) | 3.19 (1.63) | 2.21 (1.19) |
| Forced expiratory flow at 75% (L/sec) | 1.54 (0.71) | 1.08 (0.54) |
| Forced expiratory flow at 75% / Height (L/sec/m) | 0.902 (0.41) | 0.682 (0.34) |
| Extrapolated volume / Forced vital capacity *100 (%) | 4.60 (2.13) | 4.89 (2.09) |
| Tidal volume (L) | 1.00 (0.55) | 0.753 (0.44) |
| Expiratory reserve volume (L) | 1.20 (0.56) | 0.890 (0.67) |
| **Lifestyle-related factors** |  |  |
| Drinking (yes vs. no) ^b^, n/a (%) | 147/1243 (11.83%) | 24/1231 (1.95%) |
| SHS (Secondhand smoke) (yes vs. no) ^c^, n/a (%) | 169/1243 (13.60%) | 124/1231 (10.07%) |
| Nut (yes vs. no) ^d^, n/a (%) | 15/1243 (1.21%) | 1/1230 (0.08%) |
| Sport (yes vs. no) ^e^, n/a (%) | 595/1242 (47.91%) | 497/1230 (40.41%) |
| Drug (yes vs. no) ^f^, n/a (%) | 12/690 (1.74%) | 11/751 (1.46%) |
| Incense (yes vs. no) ^g^, n/a (%) | 139/690 (20.14%) | 180/752 (23.94%) |
| Coffee (yes vs. no) ^h^, n/a (%) | 247/690 (35.80%) | 274/752 (36.44%) |
| Tea (yes vs. no) ^i^, n/a (%) | 311/690 (45.07%) | 244/752 (32.45%) |
| Vegetarian (yes vs. no) ^j^, n/a (%) | 14/690 (2.03%) | 36/752 (4.79%) |
| Supper (yes vs. no) ^k^, n/a (%) | 255/690 (36.96%) | 214/752 (28.46%) |
| Eating out (yes vs. no) ^l^, n/a (%) | 661/690 (95.80%) | 705/752 (93.75%) |
| Supplement (yes vs. no) ^m^, n/a (%) | 331/690 (47.97%) | 458/752 (60.90%) |
| Daily Meals (times) | 2.91 (0.34) | 2.83 (0.43) |
| **Diet preference** |  |  |
| D1 ^n^ (1-5 points) | 2.87 (1.56) | 3.55 (1.40) |
| D2 (1-5 points) | 2.84 (1.11) | 2.97 (1.19) |
| D3 (1-5 points) | 2.53 (1.02) | 2.66 (1.09) |
| D4 (1-5 points) | 3.70 (1.27) | 4.06 (1.08) |
| D5 (1-5 points) | 3.86 (0.96) | 4.06 (0.89) |
| D6 (1-5 points) | 4.28 (0.99) | 4.32 (0.98) |
| D7 (1-5 points) | 3.72 (1.25) | 3.83 (1.22) |
| D8 (1-5 points) | 4.29 (0.77) | 4.29 (0.79) |
| D9 (1-5 points) | 3.31 (1.49) | 3.09 (1.44) |
| D10 (1-5 points) | 2.65 (1.31) | 2.48 (1.32) |
| D11 (1-5 points) | 3.19 (1.66) | 2.77 (1.62) |
| D12 (1-5 points) | 3.40 (1.64) | 3.48 (1.64) |
| D13 (1-5 points) | 3.15 (1.43) | 2.92 (1.42) |
| D14 (1-5 points) | 3.03 (1.52) | 2.65 (1.54) |
| D15 (1-5 points) | 3.10 (1.42) | 2.66 (1.35) |
| D16 (1-5 points) | 1.61 (0.92) | 1.66 (0.96) |
| D17 (1-5 points) | 3.40 (1.52) | 2.95 (1.58) |

Note: Data are presented as mean (s.d.) or n/a (%) (n: the number of individuals belonging to this category; a: the total number of individuals responding to this question).

^a^ Educational attainment: Educational attainment was recorded as an integer ranging from 1 to 7, representing different levels of education. "1" represents no formal education and illiteracy, "2" represents self-taught individuals who are literate, "3" represents the completion of elementary school, "4" represents the completion of junior high school, "5" represents the completion of senior high school or vocational high school, "6" represents the completion of college or technical school, and "7" represents the completion of graduate school or higher education.

^b^ Drinking: individuals who consumed more than 150 mL of alcoholic beverages per week for at least six months when participating in the TWB.

^c^ SHS (Secondhand smoke): being exposed to an environment with secondhand smoke (someone smoking nearby) for at least five minutes in the past six months.

^d^ Nut: betel nut chewing in the past six months.

^e^ Sport: exercising for at least 30 minutes thrice a week. Exercise included leisure-time activities such as swimming, jogging, cycling, mountain climbing, dancing, weight training, etc.

^f^ Drug: taking cough syrup, sedatives, or pain relievers at least once a week within the six months before joining the TWB.

^g^ Incense: being exposed to incense burning (e.g., during worship or the use of incense powder or rings), mosquito coils (traditional, liquid electric, or electric mosquito repellent), or fragrances (such as essential oils, aromatherapy, air fresheners, sprays, or scented candles) for at least five minutes within the past year before joining the TWB.

^h^ Coffee: coffee drinking thrice a week.

^i^ Tea: consuming tea (containing tea leaves, excluding herbal teas) at least once daily within six months before joining the TWB.

^j^ Vegetarian: vegetarian diet for at least six months before joining the TWB.

^k^ Supper: eating supper within an hour before bedtime (including milk and wine).

^l^ Eating out: eating out at least once in the past month when joining the TWB.

^m^ Supplement: regularly taking vitamins, minerals, or supplements in the past month before joining the TWB.

^n^ D1-D17: These 17 questions are included in the diet-related questionnaires listed in Supplementary Table S1.

# Table S3 Best-subset selection for 7 DNA methylation-based markers (22 factors passing the partial correlation filtering serve as candidate predictors)

|  | DNA methylation-based markers | | | | | | |
| --- | --- | --- | --- | --- | --- | --- | --- |
|  | HannumEAA  14 factors | IEAA  8 factors | PhenoEAA  14 factors | GrimEAA  15 factors | DNAmPACKYRS  12 factors | DNAmPAI1  16 factors | DunedinPACE  17 factors |
| Factors |  | | | | | | |
| Chronological age | V |  | V |  | V | V | V |
| Sex | V | V | V | V | V | V |  |
| BMI | V |  | V | V | V | V | V |
| WHR | V |  | V | V | V | V | V |
| Smoking | V | V | V | V | V | V | V |
| SBP |  |  |  |  |  | V | V |
| DBP | V | V | V |  |  |  |  |
| Fasting glucose | V |  |  |  |  | V | V |
| HbA1c |  |  | V | V | V | V | V |
| Triglyceride |  |  |  |  |  | V | V |
| HDL-C |  |  |  | V | V | V | V |
| SGPT |  |  |  |  |  |  |  |
| Creatinine | V |  |  | V |  |  | V |
| Uric acid |  |  | V | V |  | V |  |
| GGT |  |  | V | V | V | V | V |
| Hemoglobin | V |  | V | V |  |  | V |
| Hematocrit | V |  |  |  | V |  | V |
| B lymphocytes | V | V | V | V |  | V |  |
| Natural killer cells | V | V |  | V | V | V | V |
| CD4^+^ T cells | V | V | V | V |  |  | V |
| CD8^+^ T cells | V | V | V | V | V | V | V |
| Monocytes |  | V | V | V | V | V | V |
| Explanatory variables: Factors checked in the above model | | | | | | | |
| R square | 19.7% | 5.9% | 20.4% | 45.0% | 53.7% | 42.6% | 29.6% |
| Adjusted R-square | 19.3% | 5.6% | 19.9% | 44.7% | 53.5% | 42.2% | 29.1% |

Abbreviations: BMI, body mass index; WHR, waist-hip ratio; SBP, systolic blood pressure; DBP, diastolic blood pressure; HbA1c, hemoglobin A1c; HDL-C, high-density lipoprotein cholesterol; SGPT, serum glutamic pyruvic transaminase; GGT, gamma-glutamyl transferase.

# Table S4 The best model for HannumEAA, IEAA, PhenoEAA, DNAmPACKYRS, and DunedinPACE

|  | $\beta$ | Standard error | | 95% Confidence interval | | | | VIF | | FDR ^a^ | |  |
| --- | --- | --- | --- | --- | --- | --- | --- | --- | --- | --- | --- | --- |
| **HannumEAA (in years)** |  |  |  | |  | |  | |  | |  | |
| Chronological age (years) | -0.0371 | 0.0071 | | [-0.0511, | | -0.0231] | | 1.4180 | | 6.8E-07*** | |  |
| Sex (female vs. male) | -0.3588 | 0.1944 | | [-0.7400, | | 0.0225] | | 2.1470 | | 7.3E-02 | |  |
| BMI (kg/$m^{2}$) | 0.0519 | 0.0232 | | [0.0064, | | 0.0974] | | 1.6513 | | 3.2E-02 | |  |
| WHR | 3.7785 | 1.3272 | | [1.1759, | | 6.3810] | | 1.8318 | | 6.9E-03** | |  |
| CD8^+^ T cells (%) | -8.3290 | 1.6304 | | [-11.5261, | | -5.1318] | | 1.1267 | | 1.0E-06*** | |  |
| CD4^+^ T cells (%) | -17.3285 | 1.3785 | | [-20.0316, | | -14.6255] | | 1.2801 | | 5.8E-34*** | |  |
| Natural killer cells (%) | 18.1273 | 1.4798 | | [15.2256, | | 21.0291] | | 1.2192 | | 2.2E-32*** | |  |
| B lymphocytes (%) | -6.4031 | 2.8184 | | [-11.9299, | | -0.8764] | | 1.2820 | | 2.9E-02* | |  |
| DBP (mmHg) | 0.0158 | 0.0069 | | [0.0023, | | 0.0294] | | 1.3617 | | 2.8E-02* | |  |
| Fasting glucose (mg/dL) | 0.0061 | 0.0035 | | [-0.0008, | | 0.0130] | | 1.1223 | | 8.6E-02 | |  |
| Hemoglobin (g/dL) | -0.3938 | 0.0959 | | [-0.5818, | | -0.2058] | | 4.9890 | | 9.6E-05*** | |  |
| Hematocrit (%) | 0.0877 | 0.0313 | | [0.0263, | | 0.1492] | | 4.3775 | | 7.7E-03** | |  |
| Creatinine (mg/dL) | 0.4035 | 0.2148 | | [-0.0178, | | 0.8248] | | 1.1985 | | 6.8E-02 | |  |
| Smoking (yes vs. no) | 1.2521 | 0.2215 | | [0.8178, | | 1.6865] | | 1.1288 | | 6.7E-08*** | |  |
| **IEAA (in years)** |  |  | |  | |  | |  | |  | |  |
| Sex (female vs. male) | -1.2757 | 0.1700 | | [-1.6090, | | -0.9423] | | 1.3035 | | 4.1E-13*** | |  |
| CD8^+^ T cells (%) | 5.1438 | 1.7686 | | [1.6757, | | 8.6120] | | 1.0535 | | 5.9E-03** | |  |
| CD4^+^ T cells (%) | 4.3580 | 1.5883 | | [1.2435, | | 7.4724] | | 1.3515 | | 8.9E-03** | |  |
| Natural killer cells (%) | -3.2626 | 1.5755 | | [-6.3520, | | -0.1731] | | 1.0991 | | 4.6E-02* | |  |
| B lymphocytes (%) | -18.5297 | 3.1455 | | [-24.6977, | | -12.3616] | | 1.2686 | | 1.8E-08*** | |  |
| Monocytes (%) | -7.4997 | 3.7118 | | [-14.7784, | | -0.2211] | | 1.1804 | | 5.1E-02 | |  |
| DBP (mmHg) | 0.0287 | 0.0071 | | [0.0148, | | 0.0427] | | 1.1514 | | 1.2E-04*** | |  |
| Smoking (yes vs. no) | 0.3519 | 0.2463 | | [-0.1310, | | 0.8349] | | 1.1097 | | 1.5E-01 | |  |
| **PhenoEAA (in years)** |  |  | |  | |  | |  | |  | |  |
| Chronological age (years) | -0.0227 | 0.0089 | | [-0.0401, | | -0.0053] | | 1.2739 | | 1.5E-02* | |  |
| Sex (female vs. male) | 0.9992 | 0.2600 | | [0.4894, | | 1.5091] | | 2.2250 | | 2.7E-04*** | |  |
| BMI (kg/$m^{2}$) | 0.0932 | 0.0313 | | [0.0319, | | 0.1546] | | 1.7413 | | 4.7E-03** | |  |
| WHR | 3.7580 | 1.7560 | | [0.3146, | | 7.2013] | | 1.8565 | | 3.9E-02* | |  |
| CD8^+^ T cells (%) | -20.2567 | 2.1276 | | [-24.4288, | | -16.0846] | | 1.1115 | | 3.2E-20*** | |  |
| CD4^+^ T cells (%) | -23.5376 | 1.8722 | | [-27.2088, | | -19.8663] | | 1.3685 | | 5.8E-34*** | |  |
| B lymphocytes (%) | -21.0387 | 3.7095 | | [-28.3127, | | -13.7647] | | 1.2872 | | 6.3E-08*** | |  |
| Monocytes (%) | 13.2991 | 4.3060 | | [4.8553, | | 21.7429] | | 1.1591 | | 3.6E-03** | |  |
| DBP (mmHg) | 0.0252 | 0.0091 | | [0.0074, | | 0.0430] | | 1.3613 | | 8.2E-03** | |  |
| HbA1c (%) | 0.3938 | 0.1282 | | [0.1423, | | 0.6453] | | 1.1584 | | 3.7E-03** | |  |
| Hemoglobin (g/dL) | -0.3782 | 0.0759 | | [-0.5271, | | -0.2293] | | 1.8114 | | 1.9E-06*** | |  |
| GGT (U/L) | 0.0082 | 0.0027 | | [0.0030, | | 0.0135] | | 1.1247 | | 3.7E-03** | |  |
| Uric acid (mg/dL) | 0.1855 | 0.0787 | | [0.0312, | | 0.3398] | | 1.7082 | | 2.4E-02* | |  |
| Smoking (yes vs. no) | 1.8586 | 0.2908 | | [1.2885, | | 2.4288] | | 1.1252 | | 8.5E-10*** | |  |
| **DNAmPACKYRS (DNAm-based smoking pack-years)** | | | | | | | | | | | |  |
| Chronological age (years) | 0.2636 | 0.0116 | | [0.2409, | | 0.2864] | | 1.4153 | | 2.2E-102*** | |  |
| Sex (female vs. male) | -3.0770 | 0.2935 | | [-3.6525, | | -2.5015] | | 1.8486 | | 3.4E-24*** | |  |
| BMI (kg/$m^{2}$) | -0.0598 | 0.0368 | | [-0.1321, | | 0.0124] | | 1.5964 | | 1.1E-01 | |  |
| WHR (%) | 3.8990 | 2.1914 | | [-0.3983, | | 8.1962] | | 1.8805 | | 8.2E-02 | |  |
| CD8^+^ T cells (%) | -7.4933 | 2.6084 | | [-12.6082, | | -2.3784] | | 1.0923 | | 6.5E-03** | |  |
| Natural killer cells (%) | -7.3608 | 2.4147 | | [-12.0959, | | -2.6257] | | 1.2345 | | 3.9E-03** | |  |
| Monocytes (%) | 16.7706 | 5.1642 | | [6.6439, | | 26.8973] | | 1.0718 | | 2.2E-03** | |  |
| HbA1c (%) | 0.5477 | 0.1647 | | [0.2248, | | 0.8706] | | 1.1645 | | 1.8E-03** | |  |
| HDL-C (mg/dL) | -0.0165 | 0.0093 | | [-0.0349, | | 0.0018] | | 1.4102 | | 8.3E-02 | |  |
| Hematocrit (%) | 0.0592 | 0.0305 | | [-0.0007, | | 0.1191] | | 1.5830 | | 6.0E-02 | |  |
| GGT (U/L) | 0.0099 | 0.0035 | | [0.0030, | | 0.0168] | | 1.1038 | | 7.7E-03** | |  |
| Smoking (yes vs. no) | 13.6793 | 0.3833 | | [12.9276, | | 14.4310] | | 1.0942 | | 3.1E-222*** | |  |
| **DunedinPACE** |  |  | |  | |  | |  | |  | |  |
| Chronological age (years) | 0.0024 | 0.0002 | | [0.0020, | | 0.0028] | | 1.5815 | | 7.0E-29*** | |  |
| BMI (kg/$m^{2}$) | 0.0048 | 0.0006 | | [0.0036, | | 0.0061] | | 1.6878 | | 2.7E-13*** | |  |
| WHR (%) | 0.0518 | 0.0365 | | [-0.0197, | | 0.1234] | | 1.8730 | | 1.6E-01 | |  |
| CD8^+^ T cells (%) | -0.1797 | 0.0438 | | [-0.2655, | | -0.0938] | | 1.1023 | | 9.6E-05*** | |  |
| CD4^+^ T cells (%) | -0.3499 | 0.0363 | | [-0.4211, | | -0.2788] | | 1.2034 | | 1.1E-20*** | |  |
| Natural killer cells (%) | -0.1274 | 0.0404 | | [-0.2066, | | -0.0482] | | 1.2335 | | 3.0E-03** | |  |
| Monocytes (%) | 0.4482 | 0.0898 | | [0.2721, | | 0.6243] | | 1.1785 | | 1.8E-06*** | |  |
| SBP (mmHg) | 0.0003 | 0.0001 | | [0.0000, | | 0.0005] | | 1.4103 | | 3.2E-02* | |  |
| HbA1c (%) | 0.0176 | 0.0044 | | [0.0090, | | 0.0262] | | 3.1475 | | 1.3E-04*** | |  |
| Fasting glucose (mg/dL) | -0.0003 | 0.0002 | | [-0.0006, | | 0.0000] | | 3.1050 | | 8.6E-02 | |  |
| Triglyceride (mg/dL) | 0.0000 | 0.0000 | | [0.0000, | | 0.0001] | | 1.3567 | | 8.2E-02 | |  |
| HDL-C (mg/dL) | -0.0009 | 0.0002 | | [-0.0012, | | -0.0006] | | 1.5138 | | 1.8E-07*** | |  |
| Hemoglobin (g/dL) | -0.0135 | 0.0025 | | [-0.0185, | | -0.0086] | | 4.6319 | | 2.2E-07*** | |  |
| Hematocrit (%) | 0.0014 | 0.0009 | | [-0.0003, | | 0.0031] | | 4.4342 | | 1.1E-01 | |  |
| GGT (U/L) | 0.0001 | 0.0001 | | [0.0000, | | 0.0003] | | 1.1353 | | 1.1E-02* | |  |
| Creatinine (mg/dL) | 0.0110 | 0.0056 | | [0.0000, | | 0.0220] | | 1.1078 | | 5.9E-02 | |  |
| Smoking (yes vs. no) | 0.0774 | 0.0060 | | [0.0655, | | 0.0892] | | 1.1410 | | 5.7E-35*** | |  |

Abbreviations: VIF, variance inflation factor; FDR, false discovery rate; BMI, body mass index; WHR, waist-hip ratio; HbA1c, hemoglobin A1c; HDL-C, high-density lipoprotein cholesterol; GGT, gamma-glutamyl transferase.

FDR ^a^: Statistical significance is marked with *, **, and ***, representing an FDR less than 0.05, 0.01, and 0.001, respectively.

# Figures

**
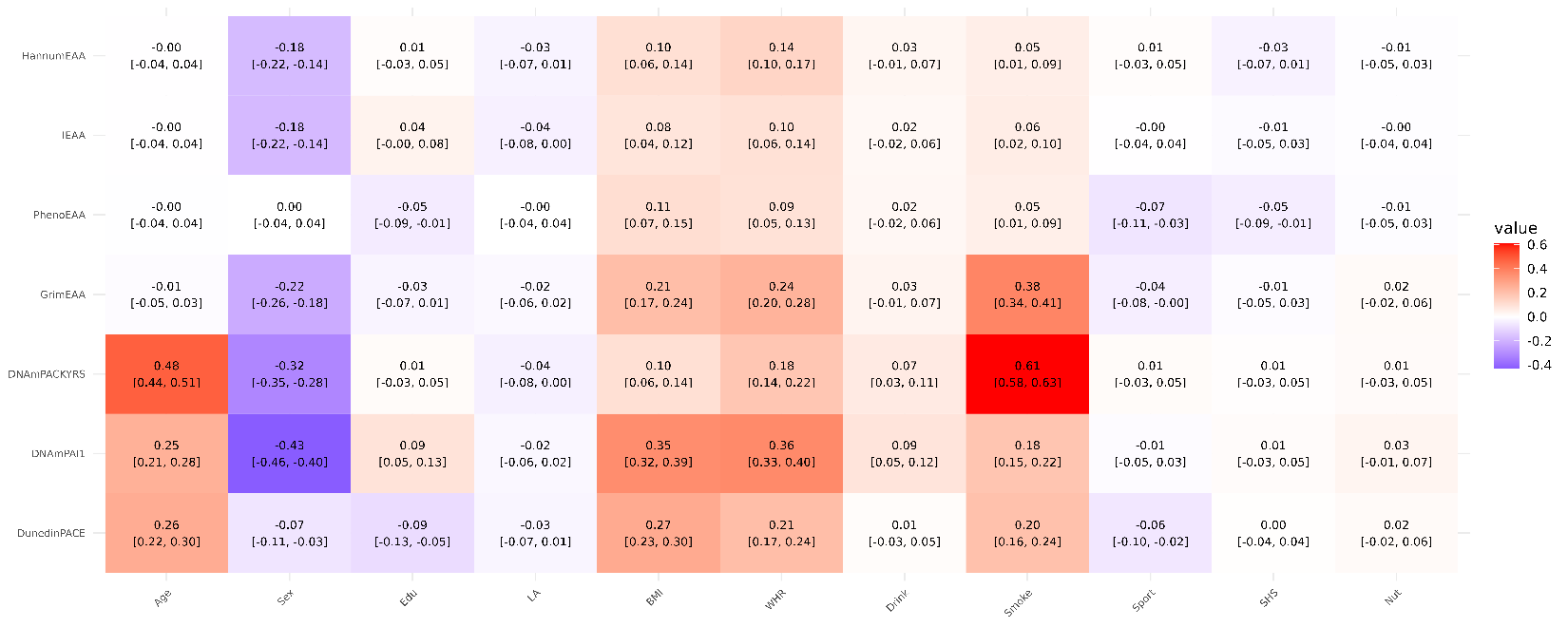
**

# Figure S1 Partial correlation analysis between DNAm-based markers and lifestyle factors

This figure illustrates the partial correlation coefficients (*r* with its 95% confidence interval) adjusted for chronological age, smoking, and drinking status, indicating the relationship between lifestyle factors and epigenetic age acceleration. Red cells denote positive correlations, while blue cells represent negative correlations.

Abbreviations: Edu, education attainment; LA, living alone; BMI, body mass index; WHR, waist-hip ratio.

Sex: 1 for males; 2 for females.

Edu (Educational attainment): Educational attainment was recorded as an integer ranging from 1 to 7, representing different levels of education. "1" represents no formal education and illiteracy, "2" represents self-taught individuals who are literate, "3" represents the completion of elementary school, "4" represents the completion of junior high school, "5" represents the completion of senior high school or vocational high school, "6" represents the completion of college or technical school, and "7" represents the completion of graduate school or higher education.

LA (Living alone): 1 for not living alone; 2 for living alone.

Drink: consuming more than 150 mL of alcoholic beverages per week for at least six months when participating in the TWB, coded as 1 = No, 2 = Yes.

Smoke: smoking cigarettes for at least six months when participating in the TWB, coded as 1 = No, 2 = Yes.

Sport: exercising for at least 30 minutes thrice a week. Exercise included leisure-time activities such as swimming, jogging, cycling, mountain climbing, dancing, weight training, etc., coded as follows: 1 = No, 2 = Yes.

SHS (Secondhand smoke): being exposed to an environment with secondhand smoke (someone smoking nearby) for at least five minutes in the past six months, coded as 1 = No, 2 = Yes.

Nut: betel nut chewing in the past six months, coded as 1 = No, 2 = Yes.


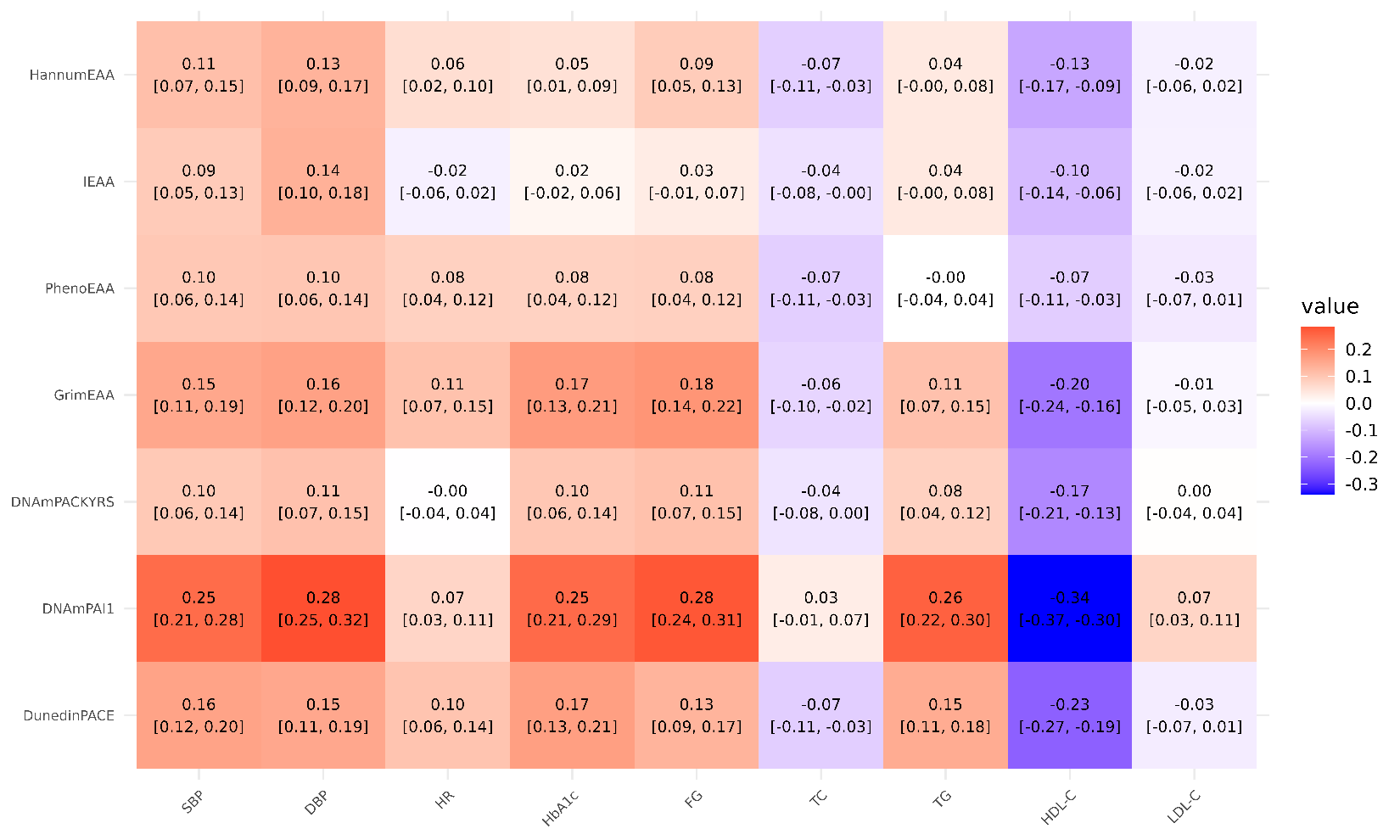


# Figure S2 Partial correlation analysis between DNAm-based markers, cardiovascular health metrics, and blood biochemical indicators

This figure illustrates the partial correlation coefficients (*r* with its 95% confidence interval) adjusted for chronological age, smoking, and drinking status, indicating the relationship between cardiovascular health metrics, blood biochemical variables, and DNA methylation age acceleration. Red cells denote positive correlations, while blue cells represent negative correlations.

Abbreviations: SBP, systolic blood pressure; DBP, diastolic blood pressure; HR, heart rate; HbA1c, hemoglobin A1c; FG, fasting glucose; TC, total cholesterol; TG, triglyceride; HDL-C, high-density lipoprotein cholesterol; LDL-C, low-density lipoprotein cholesterol.

**
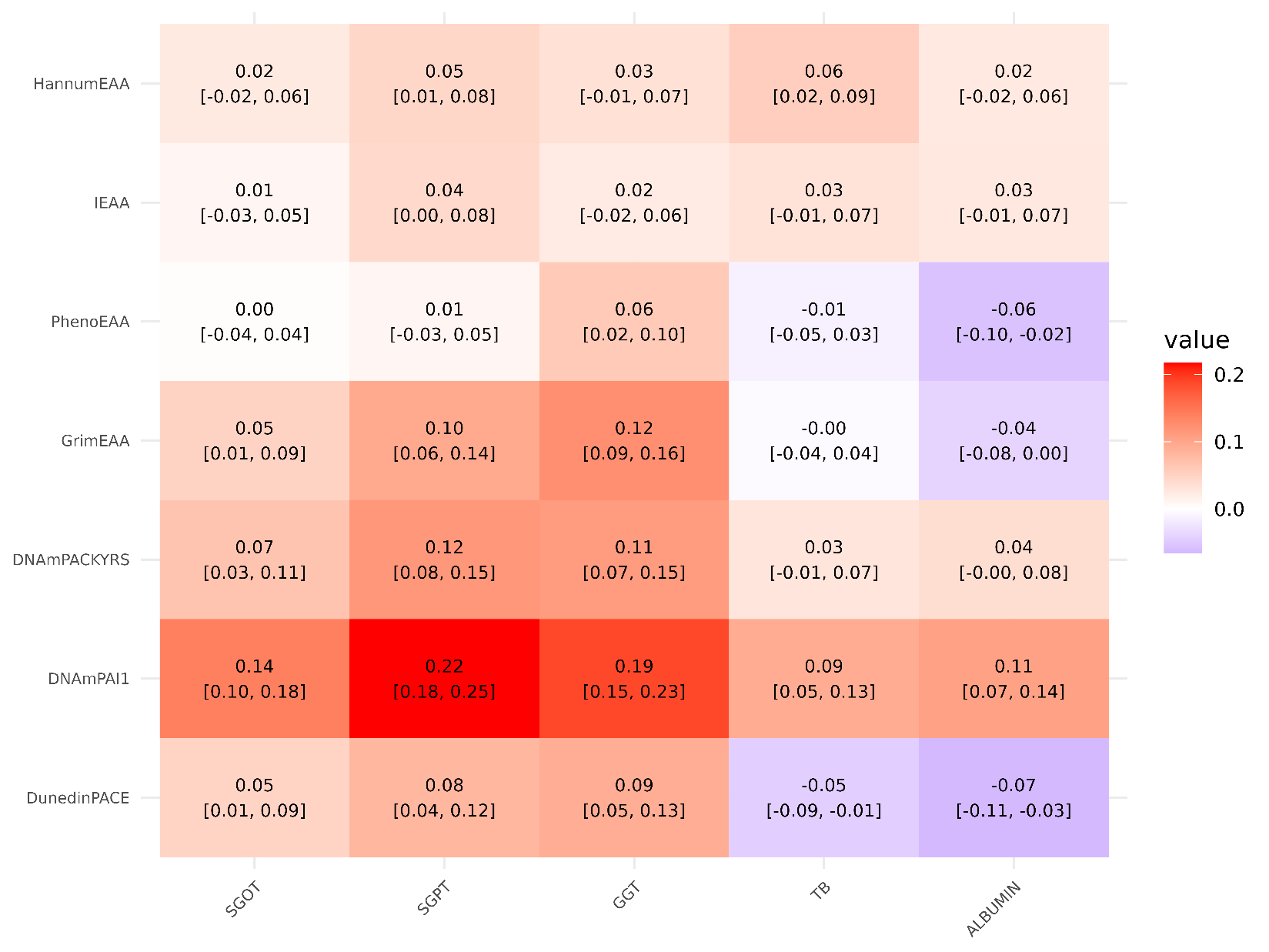
**

# Figure S3 Partial correlation analysis between DNAm-based markers and blood biochemical indicators

Partial correlation analysis was conducted between DNAm-based markers and blood biochemical variables. This figure illustrates the partial correlation coefficients (*r* with its 95% confidence interval) adjusted for chronological age, smoking, and drinking status, indicating the relationship between blood biochemical variables and DNA methylation age acceleration. Red cells denote positive correlations, while blue cells represent negative correlations.

Abbreviations: SGOT, serum glutamic oxaloacetic transaminase; SGPT, serum glutamic pyruvic transaminase; GGT, gamma-glutamyl transferase; TB, total bilirubin.


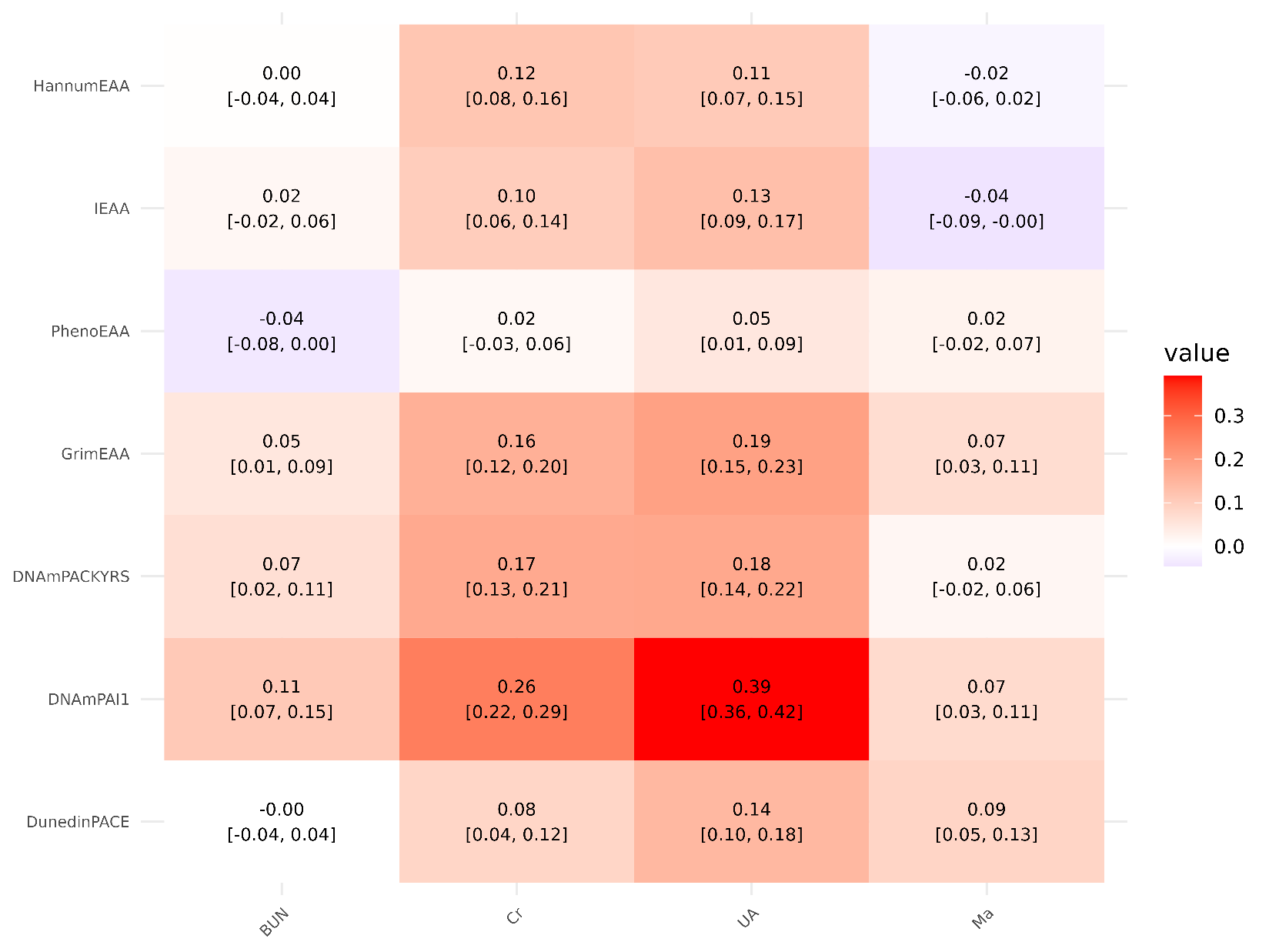


# Figure S4 Partial correlation analysis between DNAm-based markers and blood biochemical indicators

Partial correlation analysis was conducted between DNAm-based markers and blood biochemical variables. This figure illustrates the partial correlation coefficients (*r* with its 95% confidence interval) adjusted for chronological age, smoking, and drinking status, indicating the relationship between blood biochemical indicators and DNA methylation age acceleration. Red cells denote positive correlations, while blue cells represent negative correlations.

Abbreviations: BUN, blood urea nitrogen; Cr, creatinine; UA, uric acid; Ma, Microalbumin.


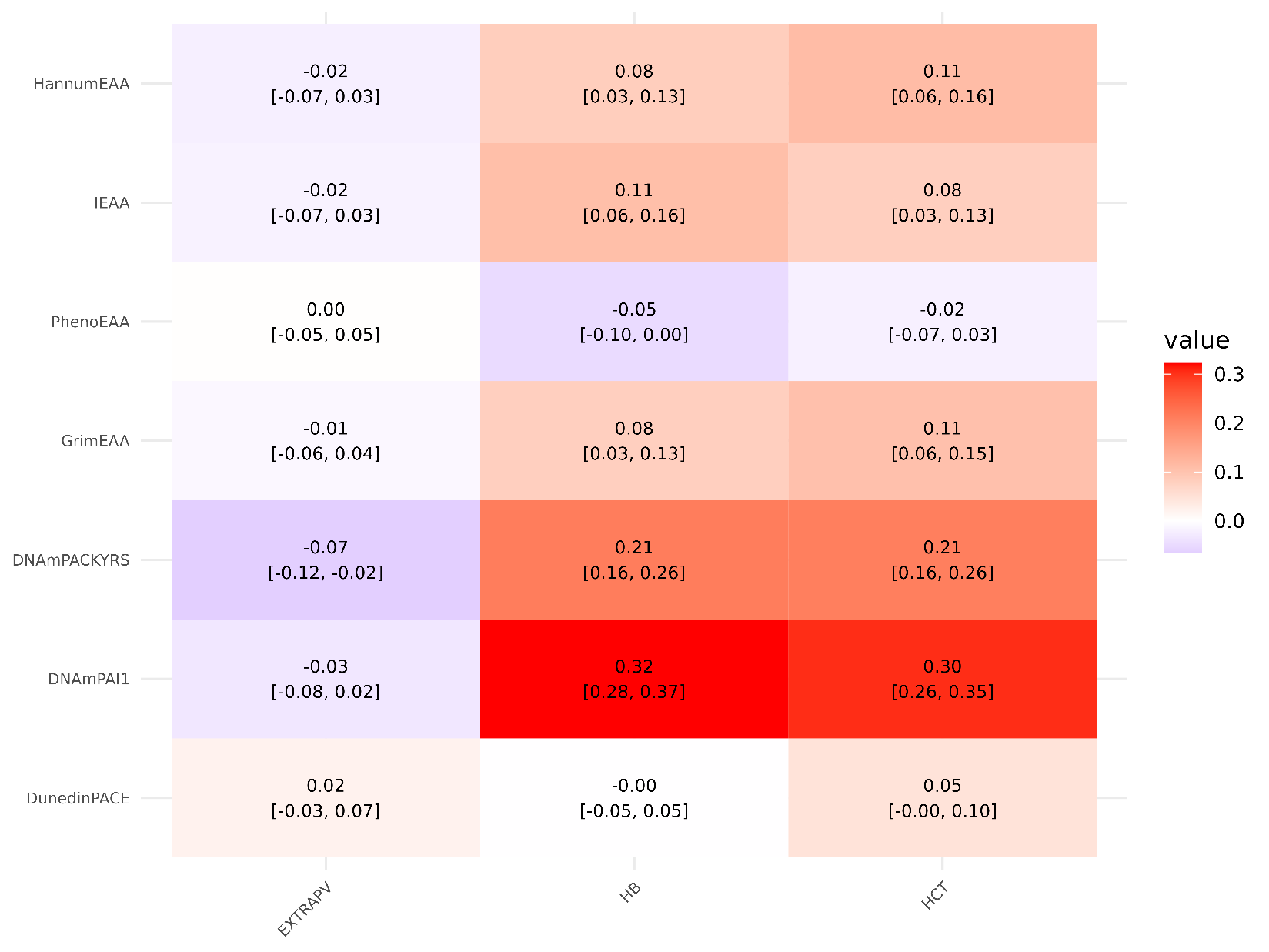


# Figure S5 Partial correlation analysis between DNAm-based markers, lung function measures, and blood biochemical indicators

This figure illustrates the partial correlation coefficients (*r* with its 95% confidence interval) adjusted for chronological age, smoking, and drinking status, indicating the relationship between lung function measures, blood biochemical indicators, and DNA methylation age acceleration. Red cells denote positive correlations, while blue cells represent negative correlations.

Abbreviations: EXTRAPV, (Extrapolated Volume/ forced vital capacity) *100; HB, hemoglobin; HCT, hematocrit.

**
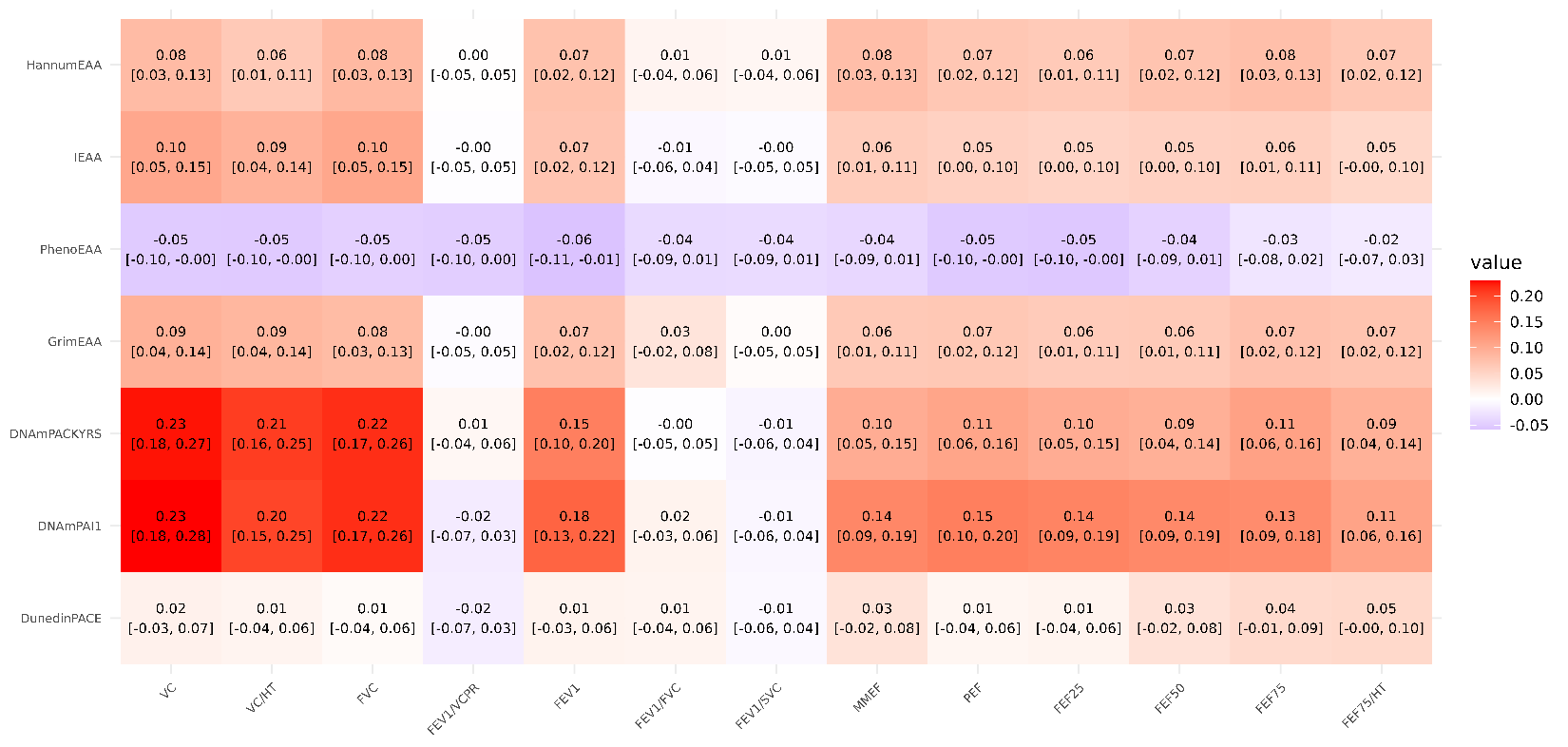
**

# Figure S6 Partial correlation analysis between DNAm-based markers and lung function measures

This figure illustrates the partial correlation coefficients (*r* with its 95% confidence interval) adjusted for chronological age, smoking, and drinking status, indicating the relationship between lung function measures and DNA methylation age acceleration. Red cells denote positive correlations, while blue cells represent negative correlations.

Abbreviations: VC, vital capacity; VC/HT, vital capacity/height; FVC, forced vital capacity; FEV1, forced expiratory volume in 1.0 s; FEV1/SVC, (forced expiratory volume in 1.0 s / vital capacity)*100; FEV1/VCPR, forced expiratory volume in 1.0 s /predicted vital capacity; MMEF, maximum mid-expiratory flow; PEF, peak expiratory flow; FEF25, forced expiratory flow at 25%; FEF50, forced expiratory flow at 50%; FEF75, forced expiratory flow at 75%; FEF75/HT, forced expiratory flow at 75%/height.


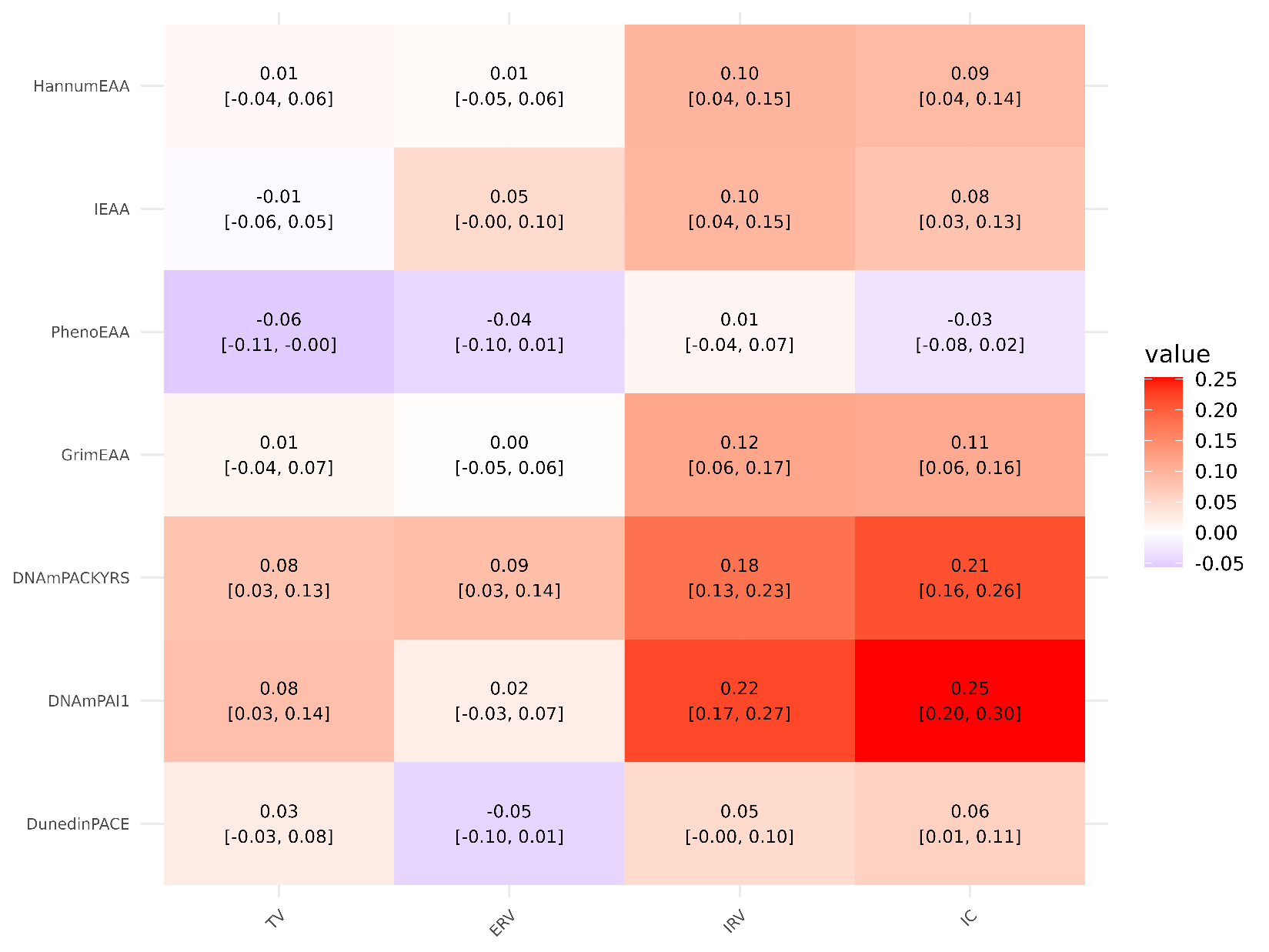


# Figure S7 Partial correlation analysis between DNAm-based markers and lung function measures

This figure illustrates the partial correlation coefficients (*r* with its 95% confidence interval) adjusted for chronological age, smoking, and drinking status, indicating the relationship between lung function measures and DNA methylation age acceleration. Red cells denote positive correlations, while blue cells represent negative correlations.

Abbreviations: TV, tidal volume; ERV, expiratory reserve volume; IRV, inspiratory reserve volume; IC, inspiratory capacity.


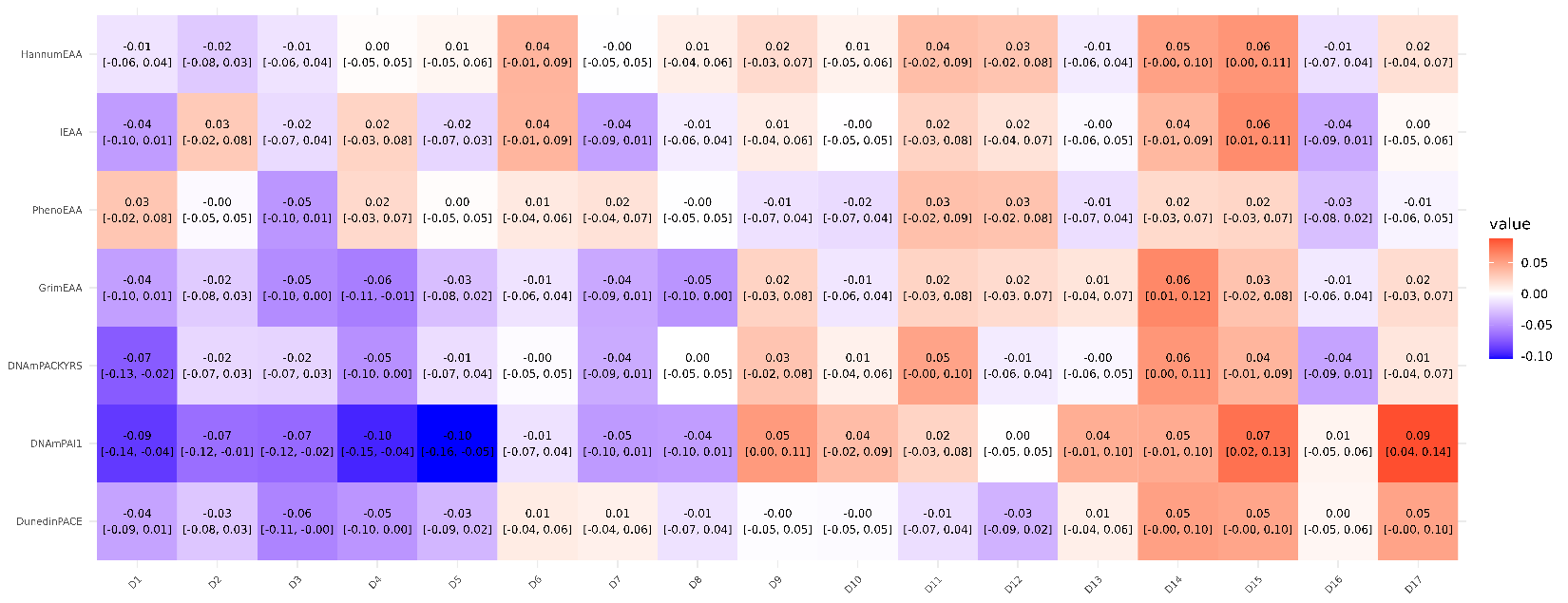


# Figure S8 Partial correlation analysis between DNAm-based markers and diet preference

This figure illustrates the partial correlation coefficients (*r* with its 95% confidence interval) adjusted for chronological age, smoking, and drinking status, indicating the relationship between diet preference and DNA methylation age acceleration. Red cells denote positive correlations, while blue cells represent negative correlations. D1-D17 are 17 questions in the TWB questionnaires, listed in the supplementary Table S1.


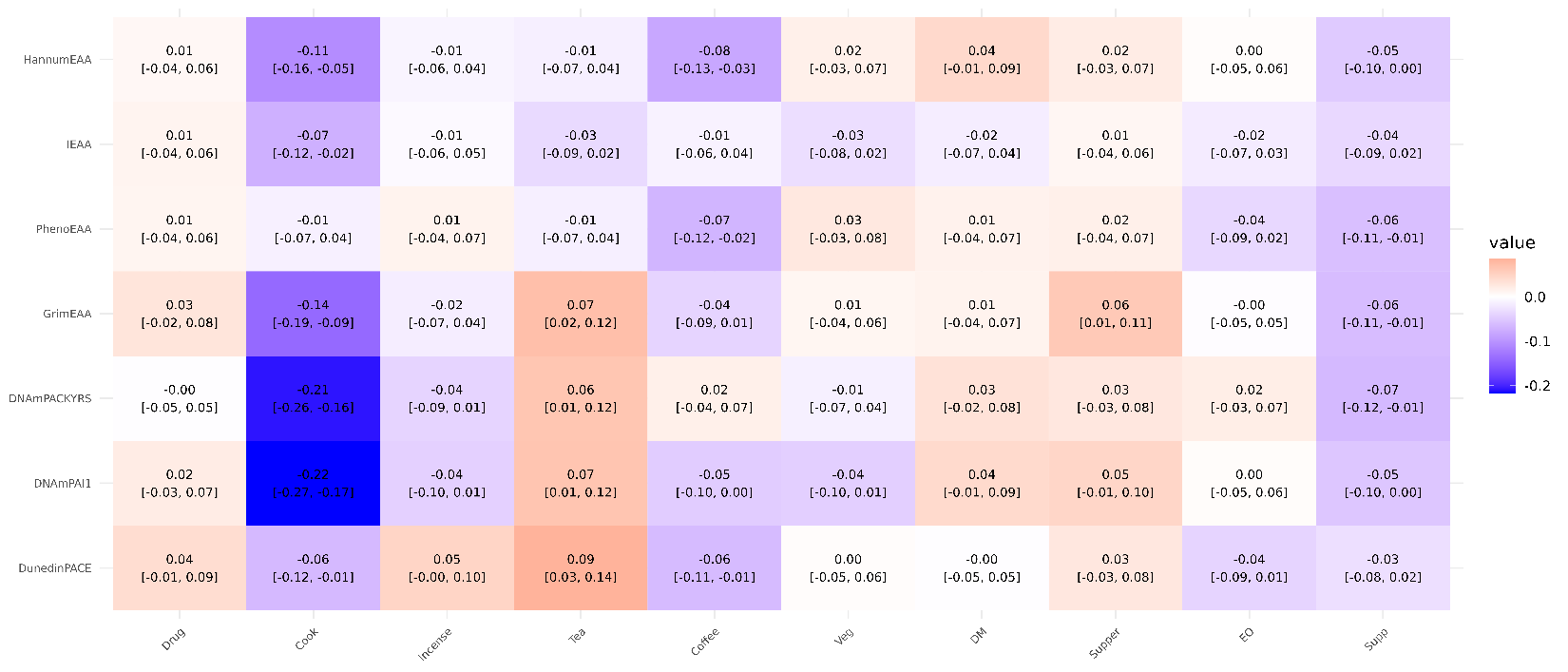


# Figure S9 Partial correlation analysis between DNAm-based markers and lifestyle-related factors

This figure illustrates the partial correlation coefficients (*r* with its 95% confidence interval) adjusted for chronological age, smoking, and drinking status, indicating the relationship between lifestyle-related factors and DNA methylation age acceleration. Red cells denote positive correlations, while blue cells represent negative correlations.

Drug: taking cough syrup, sedatives, or pain relievers at least once a week within the six months before joining the TWB, coded as 1 = No, 2 = Yes.

Cook: cooking meals by yourselves within six months before participating in the TWB, categorized as 1 = No, 2 = Yes.

Incense: being exposed to incense burning (e.g., during worship or the use of incense powder or rings), mosquito coils (traditional, liquid electric, or electric mosquito repellent), or fragrances (such as essential oils, aromatherapy, air fresheners, sprays, or scented candles) for at least five minutes within the past year before joining the TWB, categorized as 1 = No, 2 = Yes.

Tea: consuming tea (containing tea leaves, excluding herbal teas) at least once daily within six months before joining the TWB, categorized as 1 = No, 2 = Yes.

Coffee: coffee drinking thrice a week, categorized as 1 = No, 2 = Yes.

Veg: vegetarian diet for at least six months before joining the TWB, categorized as 1 = No, 2 = Yes.

DM: Daily meals, number of main meals per day, an integer ranging from 1 to 6.

Supper: eating supper within an hour before bedtime (including milk and wine), categorized as 1 = No, 2 = Yes.

EO (Eating out): eating out at least once in the past month when joining the TWB, categorized as 1 = No, 2 = Yes.

Supp: regularly taking vitamins, minerals, or supplements in the past month before joining the TWB, categorized as 1 = No, 2 = Yes.


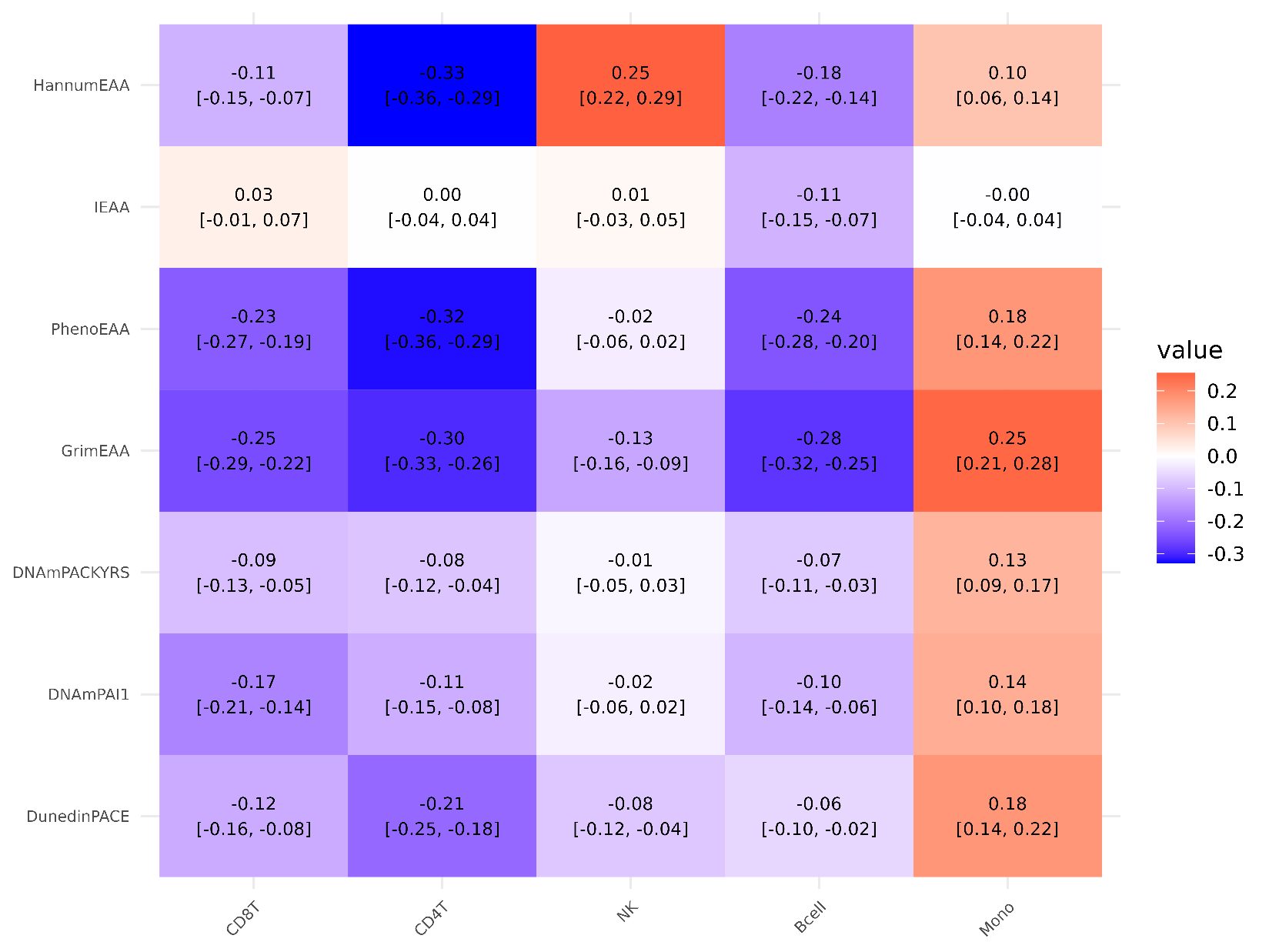


# Figure S10 Partial correlation analysis between DNAm-based markers and cell-type proportions

This figure illustrates the partial correlation coefficients (*r* with its 95% confidence interval) adjusted for chronological age, smoking, and drinking status, indicating the relationship between cell-type proportions and DNA methylation age acceleration. Red cells denote positive correlations, while blue cells represent negative correlations.

Abbreviations: CD8T, CD8^+^ T cells; CD4T, CD4^+^ T cells; NK, natural killer cells; Bcell: B lymphocytes; Mono, monocytes.

# References:

Lo, Y. H., & Lin, W. Y. (2022). Cardiovascular health and four epigenetic clocks. *Clin Epigenetics, 14*(1), 73. doi:10.1186/s13148-022-01295-7
